# Genetic foundations of interindividual neurophysiological variability

**DOI:** 10.1126/sciadv.ads7544

**Published:** 2025-07-23

**Authors:** Jason da Silva Castanheira, Jonathan Poli, Justine Y. Hansen, Bratislav Misic, Sylvain Baillet

**Affiliations:** ^1^ Montreal Neurological Institute, McGill University, Montreal, QC, Canada.; ^2^ CentraleSupélec, Université Paris-Saclay, Paris, France.

## Abstract

Neurophysiological brain activity shapes cognitive functions and individual traits. Here, we investigated the extent to which individual neurophysiological properties are genetically determined and how these adult traits align with cortical gene expression patterns across development. Using task-free magnetoencephalography in monozygotic and dizygotic twins, as well as unrelated individuals, we found that neurophysiological traits were significantly more similar between monozygotic twins, indicating a genetic influence, although individual-specific variability remained predominant. These heritable brain dynamics were mainly associated with genes involved in neurotransmission, expressed along a topographical gradient that mirrors psychological functions, including attention, planning, and emotional processes. Furthermore, the cortical expression patterns of genes associated with individual differentiation aligned most strongly with gene expression profiles observed during adulthood in previously published longitudinal datasets. These findings underscore a persistent genetic influence on neurophysiological activity, supporting individual cognitive and behavioral variability.

## Teaser

Individual brain activity profiles are shaped by lifelong genetic influences.

## INTRODUCTION

Several recent neuroimaging studies have shown that ongoing brain activity at rest, without performing a specific task, defines a neurophysiological profile unique to each person. Unlike hand fingerprints, these brain-fingerprints are associated with individual cognitive traits and are altered by pathology (*1*–*10*), forming a distinct personal neurophysiological profile. Whether a person’s genotype is associated with their neurophysiological profile is currently unknown. Heritability studies of interindividual variability (*11*) have reported some genetic associations with brain structures (*12*–*15*) and activity (*16*–*21*). Genetic factors determine, to some extent, interindividual variations in broad cognitive domains such as attentional and general intellectual abilities (*22*–*26*). Here, we studied how both individual neurophysiological and cognitive profiles relate to the spatial organization of gene expression patterns.

We used task-free magnetoencephalographic (MEG) imaging (*27*) to derive the individual neurophysiological profiles (*2*) of monozygotic (MZ) and dizygotic (DZ) twins, along with unrelated individuals. We hypothesized that if neurophysiological brain activity is determined by genetic factors, then the neurophysiological profiles of MZ twins, who share nearly identical genomes, would be nearly identical, unlike those of DZ twins (*28*). We then identified which genes are related to the features that differentiate individuals based on their neurophysiological profiles. Additionally, we investigated whether the genetic influence on these neurophysiological traits is linked to major psychological processes that characterize individual traits and how this influence aligns with cortical gene expression patterns throughout development.

## RESULTS

### Individual neurophysiological profiling and differentiation

We derived the neurophysiological profile of 89 individuals (17 pairs of MZ twins, 11 pairs of DZ twins, and 33 unrelated individuals; 22 to 35 years old) from three 6-min task-free MEG recordings provided by the Human Connectome Project (HCP) (*29*). We derived individual profiles from the distribution of neurophysiological signal power of MEG activity across the cortex for each recording (see Methods) (*2*).

**Fig. 1.**
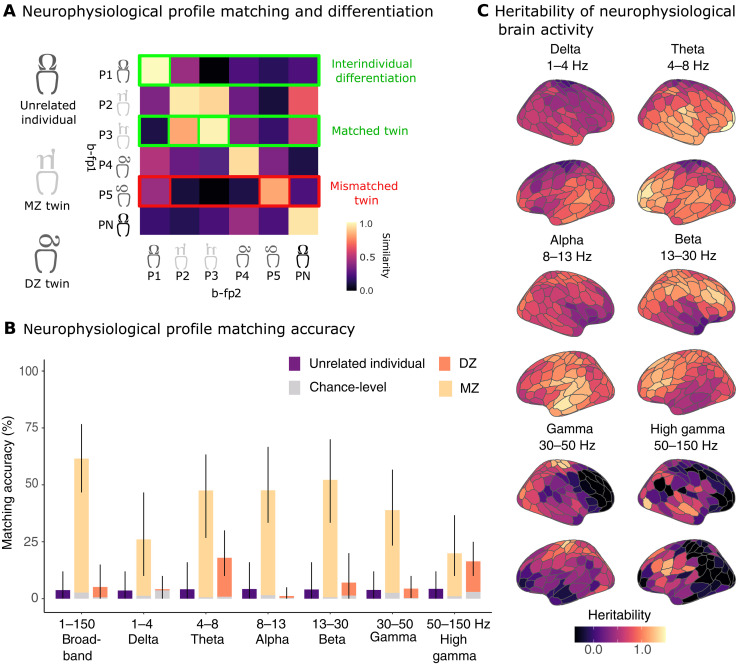
Neurophysiological profiling. (**A**) Color-coded similarity matrix showing self-similarity (diagonal elements) and between-participant similarity (off-diagonal elements) of neurophysiological profiles, computed across three independent recordings. Similarity was assessed using cross-correlation coefficients. A participant was considered correctly differentiated if their own profiles were more similar across sessions than to profiles from others. Twin sibling matching was assessed using the same similarity statistics. (**B**) Bar plots showing twin pair matching accuracies across broadband (1 to 150 Hz) and standard electrophysiological frequency bands (delta (1 to 4 Hz), theta (4 to 8 Hz), alpha (8 to 13 Hz), beta (13 to 30 Hz), gamma (30 to 50 Hz), and high gamma (50 to 150 Hz). Orange bars represent MZ and DZ twin matching accuracies; purple bars represent matching accuracies between randomly paired unrelated individuals. Gray bars represent chance-level matching based on mock neurophysiological profiles derived from empty-room MEG recordings. Error bars denote 95% CIs. (**C**) Cortical maps of heritability estimates for neurophysiological traits, computed using Falconer’s equation, illustrating the spatial distribution of genetic contributions across the cortex. Legend: b-fp1: first brain-fingerprint; b-fp2: second brain-fingerprint; MZ: monozygotic twins; DZ: dizygotic twins.

We first assessed the accuracy of interindividual differentiation based on the neurophysiological profiles obtained from the three recordings of each participant (Fig. 1). The accuracy of interindividual differentiation from these neurophysiological profiles was 83.4% [95% bootstrapped confidence interval (CI) [73.8, 90.0]; fig. S1] across a broad frequency spectrum of brain activity (1 to 150 Hz). The high temporal resolution of the data enabled us to study how the accuracy of neurophysiological profiling varied between the typical frequency bands of electrophysiology. Interindividual differentiation varied substantially across these frequency ranges, from 59.7% in the delta band (1 to 4 Hz; [57.5, 73.8]) to 87.4% in the high-gamma band (50 to 150 Hz; [80.0, 92.5]) as detailed in fig. S1.

We then evaluated the similarity between the neurophysiological profiles of siblings in a twin pair (Fig. 1A), finding that those of MZ twins matched with 61.5% accuracy ([46.7, 76.7]; Fig. 1B). In contrast, the profiles of DZ twins showed a considerably lower match accuracy of only 5.2% ([0.0, 15.0]; Fig. 1B). These differences in matching accuracies between MZ and DZ twins varied across the frequency bands of neurophysiological brain activity, as shown in Fig. 1B. The discrepancies were particularly pronounced in the alpha band (8 to 13 Hz), where MZ twins matched with an accuracy of 47.8% ([33.3, 66.7]) compared to only 1.2% for DZ twins ([0.0, 5.0]). In the beta band (13 to 30 Hz), accuracies were 52.1% for MZ twins ([33.3, 70.0]) versus 7.1% for DZ twins ([0.0, 20.0]; Fig. 1B).

To benchmark chance-level matching performance, we randomly created pairs of unrelated individuals and computed their neurophysiological profile matching accuracies, repeating this procedure 300 times per frequency band. Across all bands, matching unrelated individuals yielded low accuracy (<5%; Fig. 1B), comparable to the matching accuracy observed for DZ twin pairs. In contrast, MZ twin pairs exhibited significantly higher matching accuracies for broadband (1 to 150 Hz), theta (4 to 8 Hz), alpha (8 to 13 Hz), beta (13 to 30 Hz), and gamma (30 to 50 Hz) neurophysiological profiles (Fig. 1B). These results confirm that while neurophysiological profiles are more similar within MZ twin pairs, DZ twin similarity is not distinguishable from unrelated individuals.

To ensure the robustness of our results, we computed chance-level neurophysiological profiling and twin pair matching based on environmental and equipment noise using empty-room recordings taken before each MEG recording session (Fig. 1B). We observed above-chance differentiation across all frequency bands, including high frequencies, confirming that differentiation effects were not driven by signal-to-noise ratio (SNR) biases. We removed the variance associated with physiological artifacts from the neurophysiological profiles and found that our results remained robust (fig. S2).

Refer to fig. S3 for the distributions of self-, other-, and twin-pair similarity between neurophysiological profiles. While differentiation accuracy remains our primary metric, we provide additional analyses illustrating that MZ twins exhibit higher neurophysiological profile similarity than DZ twins, while unrelated individuals do not significantly differ from either group. These findings reinforce that differentiation accuracy is a robust measure of individual distinctiveness in neurophysiology.

### Heritability of neurophysiological traits

Although we reported above that the neurophysiological profiles of MZ twins match each other more closely than those of the general population (Fig. 1B, yellow bars), the neurophysiological profile of each MZ twin remains distinguishable from their sibling such that we can still correctly differentiate individual twins from a cohort that includes their sibling (fig. S1, purple bars). One possible explanation is that individual-specific features stand apart from the heritable aspects of their neurophysiological profile. We therefore sought to confirm that the most differentiable features of neurophysiological profiles are similarly heritable.

To assess this, we measured the spatial alignment between the most salient features for participant differentiation (fig. S4, A and B) and the heritability of neurophysiological traits (Fig. 1C and fig. S4C). While absolute heritability values provide an upper bound on genetic influence, our primary focus was on spatial patterns of heritability across frequency bands and cortical regions.

First, we leveraged intraclass correlation (ICC) statistics (*2*, *3*, *30*) to quantify which cortical parcel and frequency band contributed the most toward participant differentiation (see Methods). ICC measures the ratio of within-participant to between-participant variance, where higher ICC values indicate that a neurophysiological trait can robustly differentiate individuals across multiple recordings. Our analysis revealed that posterior cortical activity in the theta (4 to 8 Hz; ICC = 0.74), alpha (8 to 13 Hz; ICC = 0.83), and beta (13 to 30 Hz; ICC = 0.74) frequency bands were the most distinctive traits for individual differentiation (fig. S4B).

Next, we quantified the heritability of these neurophysiological traits using Falconer’s method. We observed mean heritability (H^2^) values of 0.85 for theta-band traits, 0.76 for alpha-band traits, and 0.77 for beta-band traits across the temporal, frontal, and parietal-occipital cortices (Fig. 1C). The occipital visual regions showed the highest heritability, whereas the limbic network showed the lowest (Fig. 1C). Permutation tests confirmed significant cortical-wide heritability in the alpha band and regionally significant heritability in frontal and parietal areas for beta-band traits and frontal regions for theta-band traits (fig. S5).

Last, we observed significant spatial correlations between ICC-derived differentiation maps and heritability maps, confirming that the most distinctive features of neurophysiological profiles tend to be highly heritable. These correlations were significant across broadband cortical signals (1 to 150 Hz; *r* = 0.28, *P*_spin_ = 0.026), with particularly strong relationships in the alpha (*r* = 0.62, *P*_spin_ = 0.0009) and beta (*r* = 0.58, *P*_spin_ = 0.0009) bands (fig. S6 left panel). These findings confirm that the most distinctive features of neurophysiological profiles tend to be heritable, further emphasizing the genetic basis for the neurophysiological characteristics that distinguish individuals.

### Person-specific neurophysiological signals are aligned with cortical gene expression

**Fig. 2.**
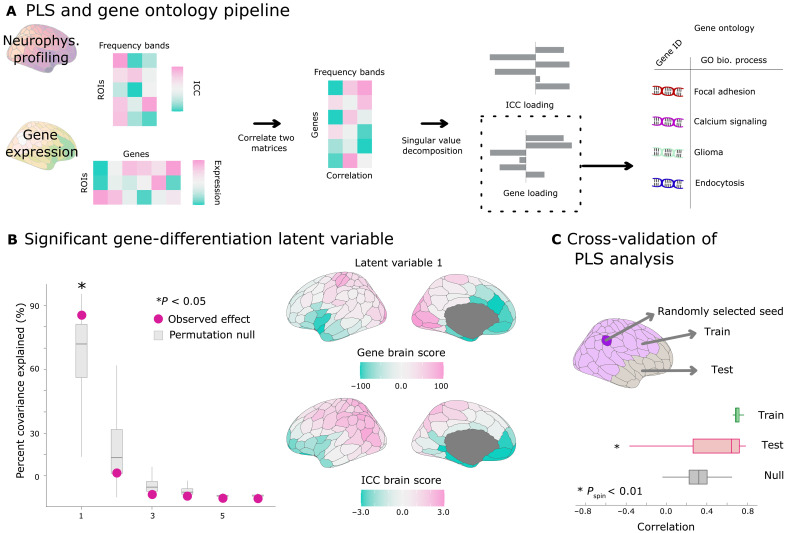
Analysis pipeline and outcomes of gene-differentiation PLS analysis. (**A**) Two data matrices were submitted to a PLS and GO analysis: (i) the first data matrix gathered the most salient traits for neurophysiological profiling and (ii) the second data matrix contained scores of gene expression across the regions of the Schaefer 200 cortical atlas (*79*). The PLS analysis resulted in latent components capturing the modes of largest covariance between these variables. Using the elements with top loadings, we performed a GO analysis to determine whether the contributing genes were enriched for specific molecular processes. (**B**) Left panel shows the PLS latent components with pink dots, ordered by decreasing effect size. Statistical significance was determined with 1000 permutations of the observed data, with spatial autocorrelation correction applied, highlighting only the first latent component. The right panel shows the related cortical topographies of gene-expression and ICC scores derived by projecting this first latent component onto the observed data. Positive gene brain scores positively covary with ICC brain scores and negatively covary with negative ICC brain scores. (**C**) We trained the PLS model using 75% of the cortical regions, selected based on their proximity in Euclidean distance to a randomly selected seed (dark purple regions), and tested the relationship between gene expression and ICC scores on the rest of the data. The median out-of-sample relationship observed was *r* = 0.64 (*P*_spin_ = 0.002).

We then assessed whether neurophysiological profiles for participant differentiation (full ICC maps from fig. S4) also align topographically with genetic cortical expressions. To do this, we studied the spatial covariation of the ICC of neurophysiological profiles with maps of cortical gene expressions with greater differential stability (>0.1) (*31*–*35*), retrieved from the microarray Allen Human Brain Atlas (AHBA) (*33*), using partial least square (PLS) correlation (Fig. 2A). We found a single PLS component significantly accounting for 85.2% of the covariance (CI [73.4, 90.1], *P*_spin_ = 0.01; Fig. 2B). This analysis revealed a topographical pattern where visual and somatomotor regions exhibited positive covariance with genetic expressions, while limbic regions exhibited negative covariance (Fig. 2B). This indicates that the most differentiable traits of individual neurophysiological profiles are spatially aligned with specific gene expression patterns along the cortical surface. We cross-validated this observation with 1000 permutations corrected for spatial autocorrelation, resulting in a median out-of-sample correlation of *r* = 0.64 (*P*_spin_ = 0.002; Fig. 2C).

We then measured how each frequency range of cortical activity contributed to this alignment between cortical gene expression and the salient traits of neurophysiological profiles. To do so, we computed the loadings for each frequency band, which correspond to the Pearson’s correlation coefficients between ICC data and the observed cortical score pattern (see Methods). We found that all frequency ranges contributed exclusively positively to this association: delta (1 to 4 Hz; *r* = 0.52, 95% CI [0.45, 0.61]), alpha (8 to 13 Hz; *r* = 0.63, 95% CI [0.58, 0.69]), beta (13 to 30 Hz; *r* = 0.52, 95% CI [0.42, 0.62]), gamma (30 to 50 Hz; *r* = 0.71, 95% CI [0.66, 0.76]), and high gamma bands (50 to 150 Hz; *r* = 0.43, 95% CI [0.34, 0.53]) with the exception of the theta band (4 to 8 Hz; r = 0.07, 95% CI [−0.09, 0.22]).

### Genes and cell types associated with neurophysiological traits

We then investigated which genes’ expressions contribute the most to the reported association with neurophysiological traits, aiming to identify the biological functions associated with these genes and the specific cell types involved. We selected the top 2208 genes based on their highest positive loadings in the PLS analysis and the top 2344 genes based on their highest negative loadings. We performed a Gene Ontology (GO) analysis using the ShinyGO pipeline and resources from the GO database (*36*), which revealed distinct biological processes linked to these sets of genes (see Methods).

**Fig. 3.**
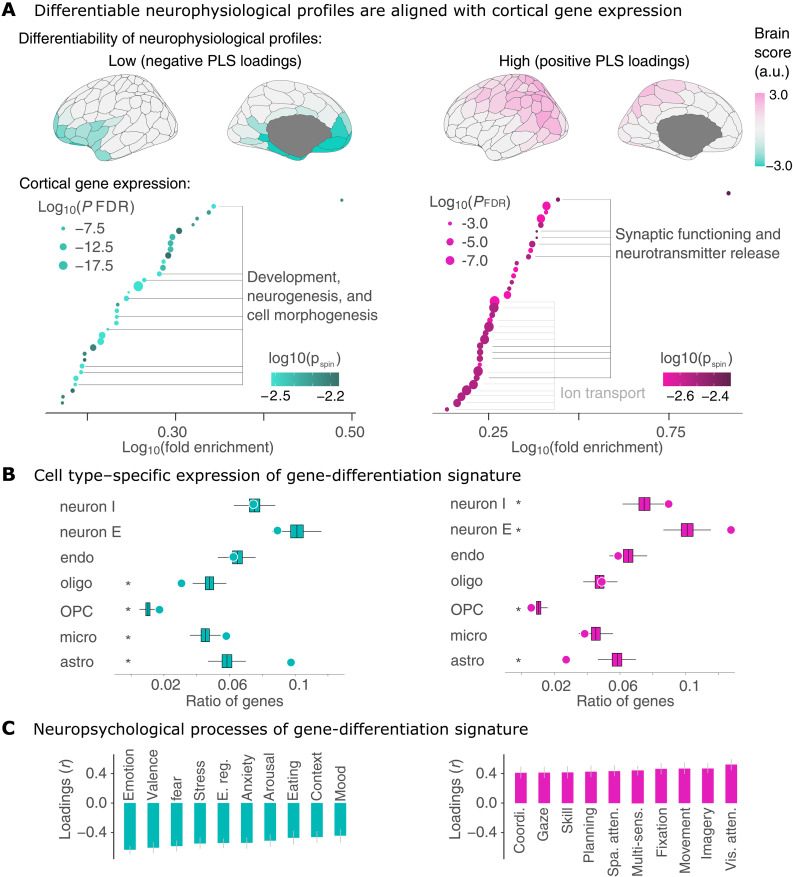
Associations between neurophysiological traits, cortical gene expression, and neuropsychological processes. (**A**) Gene-differentiation PLS analysis. The top panel shows neurophysiological brain score patterns for positive and negative loadings, indicating which cortical parcels align positively and negatively with the observed covariance pattern. The bottom panel presents the results of the GO analysis. Each point represents an enriched biological process within the corresponding gene set. The size of each point denotes the associated *P* value, while color intensity reflects the *P* value after spatial autocorrelation correction (*P*_spin_). For clarity, related terms have been grouped together using horizontal bars. (**B**) Cell-type deconvolution analysis illustrating the proportion of genes (both positive and negative) preferentially expressed in seven distinct cell types based on prior single-cell and single-nucleus RNA sequencing studies (*37*–*41*, *52*). The significance of these ratios was assessed via permutation testing (*P* < 0.05). Points represent observed ratios, while box plots show the distribution of permuted gene set ratios. Key: “neuron I” refers to inhibitory neurons, “neuron E” to excitatory neurons, “endo” to endothelial cells, “oligo” to oligodendrocytes, “OPC” to oligodendrocyte precursor cells, “micro” to microglia, and “astro” to astrocytes. (**C**) Gene-neuropsychological processes PLS analysis. This panel illustrates the top 10 psychological terms that most significantly contribute negatively (cyan) and positively (pink) to the latent component identified in the gene-neuropsychological processes PLS analysis. The bar graphs display the loadings of neuropsychological terms. CIs were computed via bootstrapping and are not necessarily symmetric. Positively weighted terms positively covary with neurophysiological profiling and the positively weighted gene set. a.u., arbitrary unit.

Genes with positive loadings were enriched in biological processes such as ion transport, synaptic functioning, and neurotransmitter release, while genes with negative loadings were associated with processes such as development, neurogenesis, and cell morphogenesis (Fig. 3A, bottom; complete gene list in the Supplementary Materials). In short, positively weighted genes covary positively with participant differentiation (i.e., the positive ICC brain scores), which in turn is associated with neurochemical signaling. In addition, we verified that the spatial alignment between gene-ontological categories was not driven by the spatial autocorrelation of cortical maps through spin tests (colors of points in Fig. 3A; see Methods).

Note that positively weighted frequency bands covary positively with positively weighted genes and negatively relate to negatively weighted genes (see Methods). For example, cortical parcels with positive scores (Fig. 2B) indicate covariance between positively weighted genes (Fig. 3A) and positively weighted frequency bands, elucidating the relationship between cortical gene expression and participant differentiation.

To determine the types of cells corresponding to these genes, we analyzed gene sets that are preferentially expressed in seven cell types as determined by RNA sequencing studies (*37*–*42*). A ratio above the null-permuted values signifies that the given gene set is preferentially expressed in a specific cell type, while a ratio lower than the permuted values would indicate that the gene set is underexpressed in that cell type. We found an overrepresentation of genes with positive loadings in excitatory neurons [*P*_FDR_ = 0.002; 1000 gene permutations, two-tailed, false discovery rate (FDR): corrected for FDR] and inhibitory neurons (*P*_FDR_ = 0.006; Fig. 3B, right). Conversely, genes with positive loadings were underrepresented in astrocytes (*P*_FDR_ = 0.002) and oligodendrocyte precursor cells (OPCs; *P*_FDR_ = 0.03). Genes with negative loadings were predominantly represented in astrocytes (*P*_FDR_ = 0.002), microglia (*P*_FDR_ = 0.004), and OPCs (*P*_FDR_ = 0.002) but were less represented in oligodendrocytes (*P*_FDR_ = 0.002; Fig. 3B, left).

These findings suggest a clear dichotomy: Genes that show positive loadings and thus positively correlate with the distinctive traits of neurophysiological profiles are predominantly expressed in neurons and underexpressed in OPCs and astrocytes. In contrast, genes with negative loadings, indicating a negative correlation with these neurophysiological profiles, are more frequently expressed in neuron-supporting cells such as astrocytes and microglia. Our results are consistent with existing models of the physiological origins of MEG signals (*27*, *43*, *44*).

### Association with neuropsychological processes

Building on prior work reporting associations between brain-fingerprint features and cognitive traits (*1*–*3*, *45*, *46*) and genetic influences on interindividual variations in cognitive domains (*22*–*26*), we investigated how the gene-neurophysiological associations found in our data may relate to neuropsychological processes.

To investigate the relationship between neurophysiological differentiation and cognitive function, we used Neurosynth, a meta-analytic tool that aggregates functional magnetic resonance imaging (fMRI) activation data from more than 15,000 studies (*47*). Neurosynth generates probabilistic cortical maps, indicating the likelihood that activity in a given brain region is associated with a specific cognitive term (e.g., “memory” and “attention”). These maps do not distinguish between task-related activations and deactivations, instead reflecting the overall spatial distribution of reported cognitive associations.

To assess whether cortical gene expression aligns with cognitive function, we performed a PLS analysis comparing cortical expression patterns with Neurosynth-derived cognitive term maps. This allowed us to test whether the genes linked to neurophysiological differentiation also show spatial correspondence with cognitive processes.

A single significant component accounted for 67.2% of this covariance (CI [54.7, 72.2%], *P*_spin_ = 0.002), highlighting a distinction between cognitive and emotional domains (Fig. 3C): Negative loadings were associated with processes related to emotions, mood, and arousal, whereas positive loadings were linked to attentional, planning, and multimodal sensory processes.

We found that these patterns of covariance between gene expression and psychological processes were strongly aligned with those linking gene expression to individual neurophysiological traits (*r* = 0.99, *P*_spin_ < 0.001; see also Supplementary Materials). We interpret these findings to suggest that the identified pattern of gene expression covaries similarly with neurophysiological profiling and activations of psychological processes: The positive set of genes positively covaries with participant differentiation and processes such as attention and planning tasks, whereas negatively weighted genes covary with poor participant differentiation (i.e., low ICC) and processes including emotions and mood (Fig. 3C).

### Developmental trajectory of the gene-differentiation signature

**Fig. 4.**
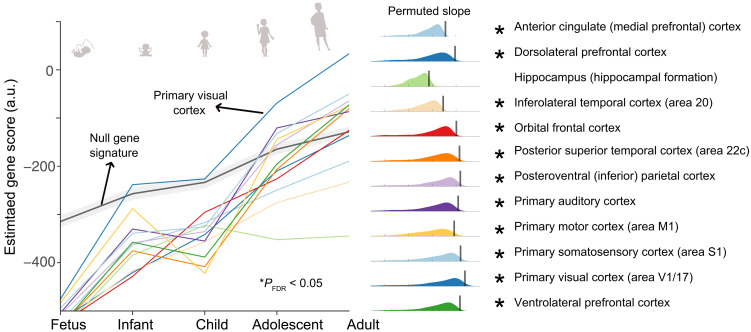
Strengthening of the gene-differentiation signature across development. The left panel illustrates the progressive increase in gene-differentiation scores across developmental stages (prenatal through adulthood) for 12 brain regions based on the BrainSpan data (*49*). A higher gene-differentiation score indicates greater similarity between the gene expression pattern at a given developmental stage and the gene-differentiation signature identified in adult neurophysiological profiles (see Fig. 3). The solid black line with gray shading indicates the trajectory of gene scores derived from random permutations of gene expression data. The right panel shows histograms of permuted slopes for each cortical region; vertical lines represent the empirical slopes, and asterisks denote regions where the gene-differentiation signature significantly strengthens across development (*P_FDR_ < 0.05). Note that this analysis compares adult neurophysiological profiles with a longitudinal dataset of cortical gene expression. It therefore does not assume longitudinal changes in neurophysiological profiles, but rather assesses whether these adult profiles align with age-related changes in gene expression patterns.

Previous work has established that genetic influences on neuropsychological processes become more pronounced with development (*24*, *26*, *48*). Following our identification of the cortical topography of a gene-differentiation signature that aligns with brain activations associated with neuropsychological processes (Fig. 3, A and C), we tested whether informative neurophysiological profiles in adulthood relate to cortical gradients of gene expression throughout development. To test this hypothesis, we assessed the topographical alignment between the cortical expression of genes across life stages in 12 cortical regions (*49*) and that of the gene-differentiation signature measured in adulthood. We found that this alignment (i.e., slope) was stronger in later life stages in all tested cortical regions, except for the hippocampus (Fig. 4). These findings suggest that individual neurophysiological profiles in adulthood are most aligned to patterns of gene expression in adulthood, which become increasingly pronounced throughout development.

## DISCUSSION

Patterns of brain activity can differentiate between individuals, akin to hand fingerprints (*1*–*3*, *50*) and are associated with neuropsychological traits (*1*, *2*, *45*, *46*) and pathophysiology (*6*, *7*, *30*). We investigated the genetic bases of individual neurophysiological profiles. Our findings indicate that genetic factors explain a significant—but not complete—portion of the variance in neurophysiological profiles. These profiles covary with a cortical gradient of gene expression enriched for neurotransmission-related genes and preferentially expressed in areas supporting higher cognitive functions. This identified gene-differentiation signature becomes progressively pronounced throughout neurodevelopment, suggesting a growing genetic contribution to interindividual variability in brain signalling.

### Heritability of neurophysiological traits

Our data show that some of the neurophysiological traits that shape individual profiles are heritable. The neurophysiological profiles of monozygotic twin pairs match each other beyond what can be explained by mere neuroanatomical resemblances (Fig. 1B and fig. S3). Conversely, our ability to match the neurophysiological profiles of dizygotic twins was at random chance, achieving similar accuracy to noise recordings (Fig. 1B). This suggests that the similarity between the neurophysiological profiles of dizygotic twin pairs is comparable to that of two unrelated individuals. These observations underscore that neurophysiological traits are in part shaped by genetics. In line with this interpretation, we observed that neurophysiological traits of alpha- and beta-band activity were heritable (Fig. 1 and figs. S5 and S6), confirming previous observations of the heritability of the individual frequency of alpha activity in humans (*16*, *17*).

The neurophysiological profiles most informative for participant differentiation followed similar spatial distributions to the heritable traits across alpha, beta, and gamma bands (figs. S4B and S6). These findings align with prior studies on MEG brain-fingerprinting, which emphasize posterior sensorimotor regions—particularly in the beta band—as key for individual differentiation (*2*, *50*, *51*).

Our study demonstrates that genetic factors play a significant role in defining the uniqueness of neurophysiological profiles, yet they do not account for all observed variations. This discrepancy suggests an explanatory gap likely attributable to environmental influences. The inability to differentiate twin pairs with absolute precision underscores the need for further research with larger twin cohorts to fully elucidate the interplay between genetics and environment in shaping individual brain activity.

### A gene-differentiation signature

Gene expression profoundly affects brain structures and functions (*32*, *52*–*57*), such as cortical folding and connectivity within brain networks (*13*, *58*, *59*). Our study extends these findings by demonstrating that, in addition to anatomical and brain-network properties, genetics also shape task-free, ongoing neurophysiological brain activity. This activity reflects a set of distinctive neurophysiological traits, uniquely defining an individual’s profile in relation to the cortical expression of specific genes, forming a gene-differentiation signature.

Using gene expression atlases, we found that these traits are related to the expression of genes regulating ion transport and neurotransmission. Genetic variants and variations in gene expression levels likely influence the function of these genes, contributing to the observed interindividual differences in neurophysiological profiles. This aligns with previous research that identified related genetic variants modulating alpha rhythms and other components of cortical neurodynamics (*16*, *18*, *60*). Our findings indicate that this gene signature is primarily active in both excitatory and inhibitory neurons (Fig. 3B). Genetic influences on these cells likely manifest as observable differences in overall brain activity patterns, leading to distinct variations in macroscale brain signaling across individuals.

Conversely, our study revealed that the cortical expression of genes involved in cell morphogenesis and neurogenesis, particularly in limbic regions, exhibits a negative correlation with individual neurophysiological differentiation (i.e., different signed loadings in the PLS analysis; see Fig. 3B). In contrast to positively loaded genes, which relate to neurochemical interactions between neurons, these negatively loaded genes are mainly expressed in supportive cells such as astrocytes and microglia. This suggests that genetic influences on neurophysiological individual traits are more related to neuronal communication than to inherited structural brain features, consistent with existing models of the physiological origins of MEG signals (*27*, *43*, *44*).

Our research highlights potential pathways for future studies on individual neurophysiology by providing a biologically grounded framework to understand behavioral variations. Animal models could be instrumental in manipulating alleles of the genes identified in our study to assess their impact on gene product functioning, large-scale brain signal characteristics, and, ultimately, behavior. This approach would provide further insights into how genetic variations influence both neurophysiological traits and behavioral differences.

### Alignment of the gene-differentiation signature with neuropsychological processes

We found that, across the cortex, the gene-differentiation signature of neurophysiological profiles aligns with maps of cortical activations related to specific cognitive and emotional processes (Fig. 3C). The spatial alignment between cortical gene expression and Neurosynth-derived cognitive maps reflects an organization pattern consistent with previously reported functional gradients (*61*–*64*). Specifically, the observed differentiation aligns with a cognitive-affective gradient, where sensory-driven cognitive processes (e.g., attention, perception) are spatially distinct from affective and emotion-related processes. These results suggest that individual differences in neurophysiology are embedded within this large-scale functional architecture.

The cortical gradients identified here—including interindividual neurophysiological differentiation, gene expression patterns, and cognitive functional associations—closely resemble the well-documented unimodal-to-transmodal axis of cortical organization (*61*–*64*). This large-scale gradient is a fundamental organizing principle of the brain, observed across multiple levels of neurobiology, including structural connectivity, functional networks, and transcriptomic variation (*62*, *64*)

Our findings suggest that interindividual variability in neurophysiological traits follows this primary axis of cortical organization. Sensory areas, which exhibit the highest neurophysiological differentiation, are functionally and molecularly distinct from transmodal association areas. These results extend prior literature by demonstrating that this organizational framework also applies to individual differences in large-scale neurophysiology. Future studies should explore the genetic and developmental factors shaping these gradients and their role in cognitive and behavioral variability.

### Trajectory of the gene-differentiation signature throughout development

Prior research has shown that genetic influences on neuropsychological processes increase across development (*24*, *26*, *48*) and that individual brain-fingerprint features become more stable and unique with age (*5*, *65*). To examine whether neurophysiological differentiation aligns with gene expression across the lifespan, we assessed the spatial alignment between adult neurophysiological differentiation maps and cortical gene expression gradients in 12 cortical regions (*49*). We found that this alignment (i.e., slope) was strongest in later developmental stages in all regions except for the hippocampus (Fig. 3).

These findings suggest that the molecular landscape supporting neurophysiological differentiation in adulthood becomes increasingly structured throughout development and provide a preliminary molecular framework for understanding age-related changes in neurophysiological variability (*4*, *66*–*70*). Our findings are supported by independent evidence demonstrating increasing genetic influences on cognition and brain activity across the lifespan (*24*, *26*, *48*). In addition, a recent complementary study on a lifespan dataset (>1000 individuals, ages 4 to 89) suggests a progressive shift from sensory to transmodal regions in individual differentiation, paralleling the developmental trajectory of gene expression gradients (*51*).

### Methodological considerations and future directions

Previous neuroimaging studies with fMRI have shown how gene expression modulates functional connectivity in the frontoparietal network, a key feature of interindividual differentiation in fMRI brain-fingerprints (*71*). In contrast, our study highlights the role of posterior unimodal sensory cortical regions in driving interindividual differentiation based on neurophysiological traits. This disparity between neuroimaging modalities underscores the distinct biological underpinnings of hemodynamic fMRI and electrophysiological signals (*1*, *2*, *50*, *72*). Specifically, while fMRI connectome-based individual profiles partially reflect the structural connections between network nodes (*73*, *74*), our findings indicate that individual neurophysiological profiles are predominantly shaped by processes of neuronal communication, particularly through the expression of genes involved in ion transport and neurotransmission. This distinction underscores the critical role of these genes in mediating neuronal signaling and brain activity patterns, providing novel insight into the mechanisms underlying neurophysiological individuality.

We must also underscore potential limitations in the interpretation of our findings. Heritability estimates were derived using Falconer’s formula, which does not account for variance due to environmental factors. Moreover, because of our limited sample size, we do not interpret heritability estimates in terms of absolute variance explained by genetics alone. Instead, we emphasize the spatial distribution of heritable neurophysiological traits and their alignment with neurophysiological differentiation patterns (figs. S4 and S6). Future studies with larger twin cohorts and comprehensive demographic documentation are warranted to refine these estimates further. Our genomics analysis is based on rare, albeit limited, data from a small sample of post-mortem brain tissue. The tissue sampling process has inherent biases, such as a focus on the left hemisphere and sex imbalance. Future research should aim to mitigate these biases. In addition, we acknowledge that post-mortem gene expression measures may not accurately reflect in vivo conditions (*75*).

A specific strength and weakness of our approach lies in aggregating data from multiple sources. While this integration uniquely enables addressing multiscale neuroscientific questions, it also presents inherent methodological challenges. To mitigate concerns regarding data aggregation (e.g., alignment between cortical maps from the AHBA and the HCP), we cross-validated our PLS results on a held-out sample of cortical parcels (Fig. 2C) and used spatial autocorrelation-preserving permutations. Our findings do not imply, however, that individual deviations in gene expression predict deviations in neurophysiology, nor do they imply direct longitudinal tracking of gene expression within individuals in the case of the BrainSpan dataset. Instead, they suggest that neurophysiological differentiation follows an organizational pattern that aligns with normative cortical gene expression. The correlational nature of our findings highlights the need for further experimental validation, potentially through animal models, to establish causative links between gene expression and neurophysiological and behavioral traits.

In conclusion, our research elucidates the relationship between molecular variations, brain activity, and individual differences. Using a multiscale, data-driven approach, the present study suggests new avenues for understanding the biological foundations of individual variability. Our findings lay the groundwork for future studies to further explore these complex interconnections, thereby enriching our understanding of the neural underpinnings of human behavior and cognition. We hope that our work will inspire continued exploration and innovation in the field, ultimately advancing our knowledge of how genetic and neurophysiological factors shape the human experience.

## METHODS

### Participants

MRI and MEG data from 89 healthy young adults (22 to 35 years old; mean = 28.6, SD = 3.8 years; see table S1) were collected from the HCP (*29*). Among these 89 participants, 34 were MZ twins, and 22 were DZ twins. The zygosity of the participants was confirmed with genotyping tests. All participants underwent three ~6-min resting-state eyes-open MEG recordings using a 248 magnetometer whole-head Magnes 3600 system (4DNeuroimaging, San Diego, CA). All sessions were conducted at the same location with a sampling rate of 2034.5 Hz, as detailed in HCP protocols (*29*). Data from twin pairs included in the HCP were sex-matched and reared similarly (*29*, *76*). This ensures that observed differences in neurophysiological differentiation between MZ and DZ twins are not attributable to differences in early-life environmental exposure.

### Ethics

The procedures for the curation and analysis were reviewed and approved according to the institutional ethics policies of McGill University ‘s and the Montreal Neurological Institute’s Research Ethics Boards (reference no. 22-06-079).

### MEG data preprocessing and source mapping

MEG data were preprocessed following good practice guidelines (*77*) using Brainstorm (*78*). Source maps for each participant’s recordings were computed using a linearly constrained minimum-variance (LCMV) beamformer and were clustered into 200 cortical parcels of the Schaefer atlas (*79*), as detailed in Supplementary Methods.

### Neurophysiological profiles

Power spectrum density (PSD) estimates at each cortical parcel were derived using Welch’s method (sliding window of 2 s, 50% overlap). The neurophysiological profile (or brain-fingerprint) of each participant consisted of PSD values defined at 301 frequency bins (range: 0 to 150 Hz; one-half–Hz resolution) for each of the 200 cortical parcels. Neurophysiological profiles were generated for each of the three MEG recordings per participant.

### Individual differentiation

Individual neurophysiological profiling was conducted following our previous work (Fig. 1A) (*2*). We assessed the correlational similarity between participants’ neurophysiological profiles across recordings. For each probe participant, we computed Pearson’s correlation coefficients between their neurophysiological profile from one of the three recordings available and a test set consisting of the neurophysiological profiles of all participants derived from another one of the other two recordings (between-participants similarity), including the probe participant’s profile (within-participant similarity). A participant was correctly differentiated if the highest correlation coefficient between their neurophysiological profile and the test set was obtained from their own neurophysiological profile from the other recording. This procedure was repeated for all participants. We then computed the percentage of correctly differentiated participants across the cohort, yielding a score of differentiation accuracy for the neurophysiological profiling approach. This procedure was repeated for all possible pairs of data recordings from the three available for each participant, and the mean differentiation accuracy was reported.

### Matching neurophysiological profiles between twin pairs

We declared that the neurophysiological profiles of twin siblings matched with one another if their Pearson’s correlation coefficients were higher than with any other participant. This matching procedure was repeated for all twin pairs in the cohort, and we reported the percentage of correctly matched pairs separately for MZ and DZ twin pairs. The results of this analysis are presented in Fig. 1B.

To test chance-level matching between neurophysiological profiles, we assessed our ability to match the neurophysiological profiles of randomly paired unrelated individuals. We randomly assigned the unrelated individuals in the cohort to another person’s neurophysiological profile and computed the matching accuracy. We repeated the randomization procedure 300 times for each frequency band.

### Band-limited neurophysiological profiles

We replicated the individual and twin pair neurophysiological profiling analyses, restricting the PSD features to those averaged over the typical electrophysiological frequency bands: delta (1 to 4 Hz), theta (4 to 8 Hz), alpha (8 to 13 Hz), beta (13 to 30 Hz), gamma (30 to 50 Hz), and high gamma (50 to 150 Hz). For example, the alpha-band neurophysiological profile consists of power spectral density estimates at each frequency within 8 to 13 Hz, rather than an average across this range.

### Bootstrapping of differentiation accuracy scores

To derive confidence intervals for the reported differentiation accuracies, we used a bootstrapping method. We randomly selected a subset of participants representing ~90% of the tested cohort (i.e., 30 MZ, 20 DZ, and 30 non-twins), derived a differentiation accuracy score, and repeated this procedure 1000 times with random subsamples of participants. We report 95% CIs from the 2.5th and 97.5th percentiles of the resulting empirical distribution of differentiation accuracies. For deriving confidence intervals for the differentiation of twin pairs, we randomly subsampled 15 MZ twin pairs and 10 DZ twin pairs for each iteration of the random subsampling.

### Saliency of neurophysiological traits

We calculated ICC using a one-way random effects model to quantify the contribution of each cortical parcel and frequency band toward differentiating between individuals across the cohort (*3*, *80*). This approach follows prior work introducing ICC for characterizing topological fingerprints in neuroimaging (*3*, *80*). ICC quantifies the ratio of within-participant to between-participant variance. High ICC values indicate the saliency of a feature of the neurophysiological profile (a neurophysiological trait) in distinguishing individuals, as it reflects high within-participant consistency and low between-participant variability. To avoid potential bias due to twin pairs, we computed ICC across all individuals in the cohort and across 100 random subsamples, ensuring only one twin from each pair was included in each subsample (i.e., for each subsample, we randomly selected either twin A or twin B to include in the calculation of ICC). The ICC values obtained from bootstrapping were nearly identical to those obtained from the entire cohort (98.6% correlation). We proceeded with the ICC values averaged across bootstraps for all analyses. The results of this analysis are presented in fig. S4B.

### Chance-level participant differentiation

To rule out the possibility that environmental factors influenced participant differentiation and twin matching accuracies, we computed a measure of chance-level differentiation and matching accuracy using mock neurophysiological profiles derived from empty-room noise recordings collected during each MEG session. These recordings, obtained from the HCP dataset (*29*), capture instrumental and environmental noise such as time of day effects (e.g., variations in background electromagnetic activity), scanner-related variability (e.g., fluctuations in baseline signal sensitivity), and external noise sources (e.g., minor variations in shielding effectiveness or room conditions).

From these empty-room noise recordings, we derived a single “mock brain-fingerprint” for each participant, based on our previous work (*2*, *8*, *51*). The noise time series was projected onto each participant’s cortical source maps, and the differentiation accuracy was recalculated using these mock profiles to test whether chance-level accuracy varied systematically across frequency bands. These were projected onto participant cortical source maps, and differentiation was recalculated using the same pipeline. The chance-level differentiation remained low (<15%) across all frequency bands, and the alpha band did not show disproportionate differentiation accuracy, ruling out SNR-driven bias.

### Heritability of neurophysiological traits

We calculated the heritability of individual neurophysiological profiles, considered as phenotypes, using the Falconer formula (*11*). This method estimates the relative contribution of genetics versus environmental factors in determining a phenotype. A phenotype in this context refers to the overall neurophysiological profile of an individual, while a trait refers to specific aspects or features within this profile, such as power spectrum density in a particular frequency band or cortical region. If the similarity in a phenotype between MZ twins is greater than that between DZ twins, the trait is considered heritable

$$H^{2}=2\left(r_{\text{MZ}}–r_{\text{DZ}}\right)$$

where *r*_MZ_ (and *r*_DZ_, respectivey) is the ICC between MZ (and DZ, respectively) twin pairs for a given neurophysiological trait. Note that heritability reflects the similarity within twin pairs for a given phenotype, whereas ICC reflects the stability of a trait within a person relative to others in the cohort.

Heritability estimates were computed using three recordings per twin, resulting in nine pairwise comparisons per twin pair (i.e., 3 × 3 comparisons) following a cross-twin cross-session pairwise comparison.

To confirm the robustness of our results, we tested whether heritability remained stable across different sample sizes and correlation metrics. Subsampling MZ twins to match DZ sample sizes resulted in highly similar heritability estimates (*r* = 0.90). In addition, using Pearson’s correlation instead of ICC produced near-identical estimates (*r* = 0.98).

### Gene expression data

Gene expression data was obtained from the six postmortem brains provided by the AHBA (http://human.brain-map.org/) (*33*) using the abagen python package (*35*). Our analyses followed a similar pipeline to prior studies (*32*). Gene expression was obtained by averaging across donors. We computed differential stability [Δ_S_(*p*)] for every gene probe. Differential stability is defined as the consistency of gene expression patterns across different donor brains, quantified using the Spearman’s rank correlation. Higher differential stability indicates that a gene exhibits spatially stable expression patterns across individuals, making it more suitable for population-level analyses. We retained 9104 genes with a differential stability above 0.1 for all future analyses, following good-practice guidelines and previous literature (*32*, *34*, *35*, *81*). See Supplementary Methods for further details.

### PLS derivation of a gene-differentiation signature

We related features for participant differentiation to gene expression gradients using PLS analysis (*82*–*84*). We *z*-scored the columns of two data arrays: one containing the full neurophysiological trait matrix (ICC values across frequency bands) and the other containing gene expression levels. The neurophysiological traits array (denoted as *Y*) had six columns representing each frequency band of interest and 200 rows representing the cortical parcels of the Schaefer atlas. The gene expression array (denoted as *X*) had 9104 columns representing genes and 200 rows representing the same cortical parcels. We applied singular value decomposition to the covariation matrix of *X* and *Y* such that

$$\text{Cov}\left(X,Y\right)=\mathrm{US}V^{T}$$

where *U* and *V* are the left and right singular vectors and *S* is the diagonal matrix of singular values. As typical with PLS, this decomposition allowed us to identify the latent variables that maximally covary between the gene expression data and the neurophysiological traits. Each singular value indicates the amount of covariance explained by its corresponding latent component. The vectors *U* and *V* provide the weights for the genes and frequency bands, respectively, for each latent component. High weights in *U* correspond to genes that strongly covary with high weights in *V*, which correspond to specific frequency bands. Positively weighted genes covary with positively weighted frequency bands, elucidating the relationship between genetic and neurophysiological variability.

Gene expression and ICC scores were computed for each cortical parcel by projecting the original data matrices *X* and *Y* onto the singular vector weights obtained from the PLS analysis. Specifically, these scores represent the covariance between the gene expression data and the neurophysiological traits. For example, cortical parcels with positive scores indicate covariance between positively weighted genes and positively weighted frequency bands, which are important for participant differentiation.

The contribution of each frequency band to the observed latent variable (i.e., loadings) was computed using Pearson’s correlation between ICC values and the cortical score pattern obtained from PLS analysis. These correlations represent the loadings for each frequency band, quantifying their alignment with the gene-differentiation signature. Loadings were computed as Pearson’s correlation coefficients between each variable’s regional spatial distribution over the cortex (i.e., gene expression and ICC data) and the corresponding cortical score pattern (i.e., correlating gene expression with ICC scores). We used Pearson’s correlation coefficients for our loadings because they provide a standardized measure of the strength and direction of the linear relationship between variables, facilitating interpretation. Variables with large absolute loadings are highly correlated with the observed score pattern and strongly relate to the latent component of covariance.

To assess the significance of the latent components, we conducted permutation tests that preserved the spatial autocorrelation of cortical maps (see below). Specifically, we performed 1000 spin tests and computed a null distribution of singular values. *P* values were computed as the proportion of null singular values that achieved a greater magnitude than the empirical singular values. In addition, we computed bootstrapped confidence intervals for the singular values by randomly resampling the rows (corresponding to the cortical parcels) of both data matrices 1000 times. We report the 2.5th and 97.5th percentiles of the resulting distribution of singular values. The results of this analysis are presented in Fig. 3A.

### Gene ontology analysis

To determine the biological processes that strongly contributed to the set of positively and negatively loaded genes, we conducted an enrichment analysis using GO (*85*, *86*), a framework for categorizing gene products based on their molecular function and associated biological processes (*36*, *85*).

For negatively and positively loaded genes, we separately selected the 50% with the largest absolute loadings (e.g., genes with the 50% most negative or positive loadings) and input these genes into the ShinyGO v.0.77 GO tool (*36*) using the GO biological processes pathway databases (*86*). ShinyGO is an accessible bioinformatics Shiny app that conducts GO analyses to identify which biological processes are enriched in any given set of genes. Genes without Enrtez IDs were excluded from the analysis. *P* values associated with fold enrichment for all terms were FDR-corrected. See Supplementary Data for a comprehensive list of all biological processes and their corresponding fold enrichment values. The results of this analysis are presented in Fig. 3C.

Previous research has shown that standard gene ontology analyses may inflate significance estimates due to spatial autocorrelation in cortical maps (*87*). To mitigate this issue, we used spatial autocorrelation-preserving permutation tests to rigorously evaluate statistical significance (see the “Correction for spatial autocorrelation of cortical maps” section). Specifically, for each GO category, we computed the spatial alignment between gene expression topographies and neurophysiological differentiation maps and then assessed significance against 1000 iterations of spatially permuted cortical maps. This approach ensures that our findings exceed what would be expected under a random spatially autocorrelated null model.

Critically, all previously reported GO categories remained significant following these rigorous control analyses, confirming the robustness of our findings. These results are now illustrated in Fig. 3A (see *P*_spin_ values).

### Cell-type deconvolution

We aggregated cell-specific gene sets for seven cell types using data from five human adult postmortem single-cell and single-nucleus RNA sequencing studies (*37*–*41*, *52*). The seven cell classes were determined based on hierarchical clustering, resulting in the following cell types: astrocytes, endothelial cells, microglia, excitatory neurons, inhibitory neurons, oligodendrocytes, and OPCs.

We assessed the preferential expression of cell-specific gene sets by (i) computing the ratio of positively loaded genes that overlapped with the cell-specific gene set and (ii) permuting gene sets 1000 times to assess statistical significance.

This approach allowed us to determine the statistical significance of the overlap between the loaded genes and the cell-specific gene sets, providing insights into the cell type–specific expression patterns of the genes contributing to the neurophysiological traits. The results of this analysis are presented in Fig. 3B.

### Gene expression and neuropsychological processes PLS analysis

We repeated the above-described PLS analysis to relate gene expression to neuropsychological processes, as indexed by brain activation maps obtained from Neurosynth (*47*). This analysis replicated the approach of Hansen and colleagues (*32*) using the Schaefer 200 atlas (*79*). For detailed methodology, see Supplementary Methods.

### Gene-signature evolution across developmental stages

We used brain gene expression data available from BrainSpan (*49*), which features gene expression levels from different developmental stages ranging from 8 post-conception weeks to 40 years of age. We computed gene scores for 12 cortical regions across neurodevelopmental stages by multiplying the gene expression matrix obtained from BrainSpan with the PLS-derived gene weights (columns of *U*) obtained from the gene-differentiation PLS analysis (see the “PLS derivation of a gene-differentiation signature” section).

We fitted linear slopes—using the MATLAB *polyfit*() function—to the gene scores across neurodevelopment for each cortical parcel separately. These slopes were then compared to statistical null slope values obtained by performing spatially autocorrelation-preserving permutations, then running the PLS analysis pipeline, and multiplying the null gene weights with BrainSpan gene expression data. This resulted in a null distribution of slopes (1000 permutations). See the Supplementary Methods for further details. The results of this analysis are presented in Fig. 3 and fig. S7.

### Visualization

We plotted brain maps of ICC, heritability, and PLS brain scores using the *ggSchaefer* and *ggseg* R packages. All other plots were generated using the *ggplot2* package in R (*88*).

### Correction for spatial autocorrelation of cortical maps

We corrected for spatial autocorrelation of cortical map data, where applicable, using spin tests. Spin tests preserve the spatial autocorrelation of cortical topographies by rotating the cortex surface data, effectively permuting the spatial positions while maintaining the spatial structure. This generates a null distribution that accounts for spatial autocorrelation. We conducted 1000 spin permutations of our brain maps using the Hungarian method (*89*, *90*).

## Supplementary Materials

### The PDF file includes:

Supplementary Materials and Methods Tables S1 and S2 Figs. S1 to S6 Legends for data files S1 to S4 References
