# Supplementary material for: Genetic foundations of interindividual neurophysiological variability
Source: Sci Adv. 2025 Jul 23;11(30):eads7544. doi: 10.1126/sciadv.ads7544 (PMC12285703; doi:10.1126/sciadv.ads7544)
Supplement: Supplementary file 1 — Supplementary Materials and Methods Tables S1 and S2 Figs. S1 to S6 Legends for data files S1 to S4 References [file sciadv.ads7544_sm.pdf]

Science Advances
AAAS

Supplementary Materials for

Genetic foundations of interindividual neurophysiological variability

Jason da Silva Castanheira *et al.*

Corresponding author: Sylvain Baillet, sylvain.baillet@mcgill.ca

Sci. Adv. **11**, eads7544 (2025)
DOI: 10.1126/sciadv.ads7544

The PDF file includes:

Supplementary Materials and Methods
Tables S1 and S2
Figs. S1 to S6
Legends for data files S1 to S4
References

Other Supplementary Material for this manuscript includes the following:

Data files S1 to S4

Supplemental Materials:

Methods (Cont'd.)

MEG data preprocessing. MEG data were preprocessed following good practice guidelines (*77*) using *Brainstorm* (*78*) March-2023 distribution running MATLAB 2020b (Mathworks Inc., Massachusetts, USA). Our preprocessing pipeline was adapted from previous published work (*2*, *30*). Line noise artifacts (60 Hz) along with their first 9 harmonics were removed using notch filters. Slow-wave and DC artifacts were attenuated with a high-pass FIR filter above 0.3 Hz. To remove ocular and cardiac physiological artifacts, we defined Signal-Space Projections (SSPs) based on the activity of concurrent electro-cardiogram and -oculogram recordings. We additionally attenuated low-frequency eye saccades (1-7 Hz) and high-frequency (40-240 Hz) muscle noise components with SSPs.

MEG source mapping. We source imaged the resting-state MEG sensor data using the coregistered anatomy folder provided by HCP (*29*). We computed MEG biophysical head models for each participant using the *Brainstorm* overlapping-spheres model (default parameters) applied to 15,000 locations distributed over the entire cortex. Source maps for each participants' recording were computed using linearly-constrained minimum-variance (LCMV) beamforming (using *Brainstorm*'s default parameters: 2018 version). Noise statistics were estimated from the empty-room recordings collected on the respective day of the visit of each participant. Individual source maps were then projected onto a default anatomy template, spatially smoothed (3mm) and clustered into the 200 cortical regions of the Schaefer atlas (*79*) using the first principal component within each region as a representative time series of brain activity. Brain-fingerprints were derived from the power spectrum densities (PSD) of these regional source time series computed using Welch's method with a sliding window of 2 seconds and 50% overlap.

Correspondence of salient neurophysiological traits and heritable brain phenotypes. We determined whether the salient features for individual differentiation were aligned topographically with heritable brain phenotypes. To do this, we computed the Pearson's spatial correlation of ICC neurophysiological profile topographies with the brain maps obtained from the heritability analyses (see **Heritability of brain phenotypes)** across the 200 regions of the Schaefer atlas(*36*). We controlled for the spatial autocorrelation of the data using the Hungarian method (*90*, *91*) (see **Correction for spatial autocorrelation of brain maps**).

Neuroanatomy. We verified that the neurophysiological profiles of MZ twin pairs matched in spite of heritable neuroanatomical features. We, therefore, derived structural statistics for each region of the Desikan-Killiany atlas from *Freesurfer* (*91*). We then i) computed the heritability of these features following the procedure described in the main text, and ii) tested for a possible linear association between anatomical and spectral similarity across twin pairs. The results are reported separately for MZ, and DZ twin pairs (see The Matching Between the Neurophysiological Profiles of Monozygotic Twins Is Not Driven by Anatomy).

Biophysical and environmental artifacts. We investigated whether MEG recording artifacts might have overly contributed to the differentiation between individuals. We computed the root-mean-squares (RMS) of ocular and cardiac reference signals (ECG, HEOG, VEOG, respectively) collected simultaneously with MEG data. We then linearly regressed these measures from the neurophysiological profiles and used the residuals of this regression to differentiate individuals.
We then tested whether the environmental and instrument noise conditions on the day of the MEG recordings biased individual differentiation (*2*). We, therefore, used the empty-room recordings collected on the same day of the MEG session for each participant to derive pseudo-neurophysiological profiles. These empty-room recordings were preprocessed using the same filters as the resting-state data and projected onto the participant's brain using the same imaging kernels. We computed the differentiation accuracies obtained based on these pseudo-profiles.

Gene expression data. Gene expression data were obtained from the six postmortem brains provided by the AHBA (http://human.brain-map.org/) (*33*) using the *abagen* Python package (*35*), following a pipeline published previously (*32*). In brief, we first used microarray probes with the highest differential stability to represent gene expression for each gene (20,232 in total). Tissue samples were assigned to each of the 200 brain regions of the Schaefer atlas using Montreal Neurological Institute (MNI) coordinates generated via nonlinear registrations. We ignored tissue samples further than 2 mm away from each brain region. To reduce potential misassignment, sample-to-region matching was constrained by hemisphere and to the cortex. If a region of the Schaefer atlas was not assigned a sample, the closest sample in Euclidian distance to the centroid of the region was selected. Gene expression was normalized across tissue samples and subjects, and for each of the retained genes, was obtained by averaging across donors. We retained 9104 genes with a differential stability above 0.1 in further analyses, following good-practice guidelines and previous literature (*32*, *34*, *35*, *81*).

Cross-validation of gene-differentiation PLS analysis. We assessed the robustness of our PLS model through cross-validation of Pearson's correlation between the observed gene scores and ICC statistics. We followed the same cross-validation procedures as Hansen and colleagues (*32*), splitting brain regions into one testing and one training set. A random seed was used to determine the training set: 75% of the brain regions the closest in Euclidian distance to the seed location were used to train the PLS model. The quartile of regions were held out to test the PLS model by computing the correlation between predicted gene scores and ICC statistics [*Corr(XtestUtrain, YtestVtrain*)]. This procedure was repeated 100 times to produce a distribution of correlations. The significance of the cross-validation outcomes was assessed against a null model obtained from spatial autocorrelation-preserving permutations of the gene expression matrix and repeated the cross-validation procedure 1,000 times (Figure S7c).

Gene expression & psychological-processes PLS analysis. We assessed the relationship between gene expression and psychological processes as indexed by brain activation maps obtained from *Neurosynth* (*47*).

The brain map associated with each psychological-process term represents the probabilistic association between this term (e.g., attention) and brain activations observed at each voxel from

published studies reporting on that psychological process. This meta-analytic approach combines data from >14,000 published fMRI studies. We focused our analyses on the 123 terms reported by Hansen and colleagues (*32*) at the intersection between Neurosynth (*47*) and the Cognitive Atlas (*92*), a public ontology of cognitive science. This data-driven approach did not distinguish between activations and deactivations, nor did it consider the degree of activation of a given brain area. Here too, we used the Schaefer-200 atlas (*79*) to sample the resulting cortical maps.

We assessed the alignment between the respective latent components associated with gene-psychological processes and gene-differentiation by computing the Pearson's correlation between gene scores and PLS loadings (see Psychological Processes and Differentiation).

Gene ontology analysis. To determine the biological processes contributing to positively and negatively loaded genes, we performed an enrichment analysis for the 50% largest loadings (e.g., genes with the 50% most negative and positive loadings) using the *ShinyGO* V 0.77 (Dec 20th 2023) gene ontology tool (*36*) and the GO pathway databases of biological processes (*86*). Genes with no Entrez Ids were ignored. Fold enrichment for each biological process was computed by comparing the frequency of a given biological process in the set of positive genes to the frequency of that process in the entire genome. P-values associated with fold enrichment for all terms were corrected for false discovery rate (FDR). See Supplemental Data for a comprehensive list of all biological processes and their corresponding fold enrichment values.

Development of the gene signature. We binned gene expression data from BrainSpan (*51*) into five life stages: fetal (8–37 post-conception weeks), infant (4 months–1 year), child (2–8 years), adolescent (11–19 years) and adult (21–40 years) (*93*). For each life stage, we computed the gene expression of the top 50% of positively and negatively loaded genes for each cortical region. Additionally, we computed gene expression at every neurodevelopmental stage for a random set of genes (Figure S7B). Note that of the 16 cortical regions with gene expression data, four regions only had samples for the fetal stage; therefore, we report data for the 12 cortical regions with data across all neurodevelopmental stages.

Supplementary Results

	Age (mean ± SD)	Sex (F/M)	Education (mean ± SD)
Non-twins)	29.08 ± 3.30	13/12	13.8 ± 2.16
Monozygotic	27.45 ± 3.94	16/22	15.31 ± 1.34
Dizygotic	29.85 ± 3.87	12/14	15.00 ± 1.57

Table S1: Demographic information.

Assessing the Robustness of Neurophysiological Profiles

We performed a series of sensitivity analyses to rule out the possibility of environmental and physiological artifacts affecting our results.

We first evaluated the influence of environmental factors that may affected the MEG recordings. We processed empty-room recordings in the same way as the actual participant data to derive pseudo-neurophysiological profiles related to the environmental conditions around each participant's visit. Individual differentiation was poor based on these pseudo-neurophysiological profiles (<1.7%; Figures 1B).

We then used linear regression models to remove the variance associated with physiological artifacts from the neurophysiological profiles (see Methods (Cont'd.) **Biophysical and environmental artifacts**). Using the same analysis pipeline, we observed that identification accuracy remained largely unaffected: 82.6% [74.7, 89.3] differentiation accuracy across all participants, 55.2% [46.7, 66.7] matching accuracy between monozygotic twins, and 5.8% [0.0, 15.0] between dizygotic twins, using broadband features (1-150Hz; Figure S2). The robustness of the results indicates that individual differentiation is not significantly driven by physiological artefacts.

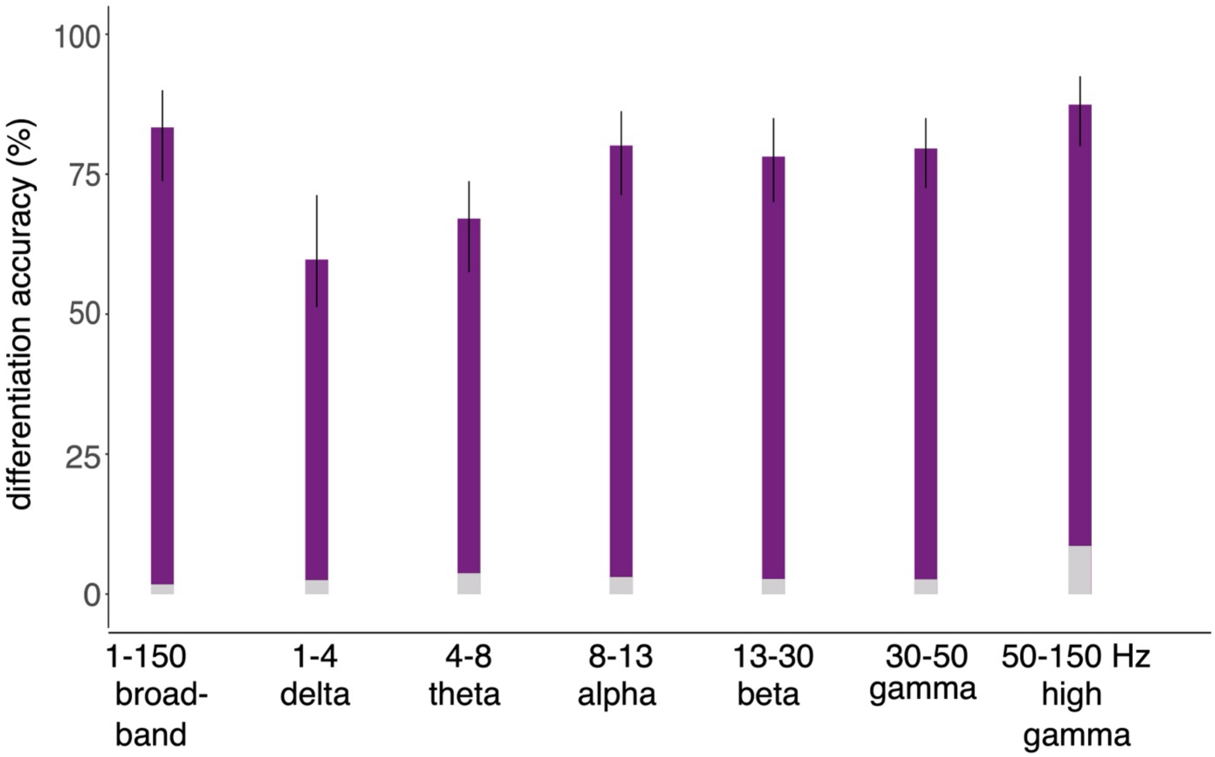

Figure S1. Participant differentiation accuracy.
The differentiation accuracy scores of neurophysiological profiles for all individuals. Participants can be accurately differentiated from neurophysiological profiles across all frequency bands, principally the alpha, beta, gamma, and high gamma bands. Grey bars at the foot of each plot indicate Chance-level participant differentiation accuracy computed using 'mock brain-fingerprints' derived from environmental and instrument noise recorded during MEG sessions. Error bars represent 95% confidence intervals.

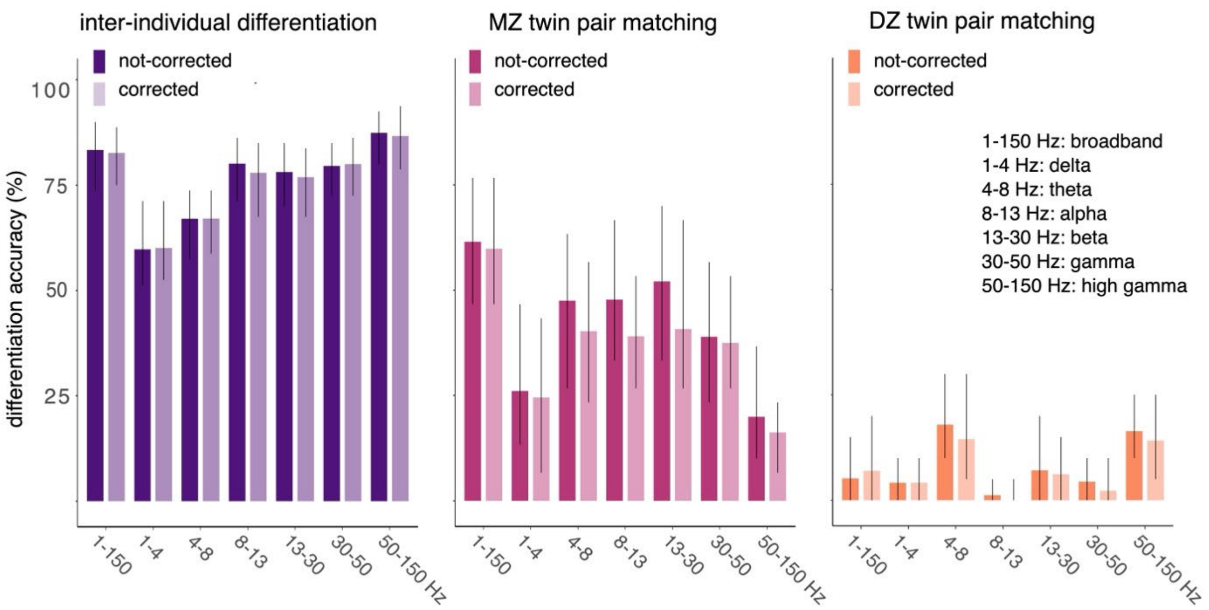

Figure S2. Physiological artifacts do not impact differentiation & matching accuracy. Comparison of the differentiation and twin-matching accuracy scores before and after regressing out the influence of artifacts on neurophysiological profiles, for singletons (left panel), MZ twin pairs (middle panel) and DZ twin pairs (right panel).

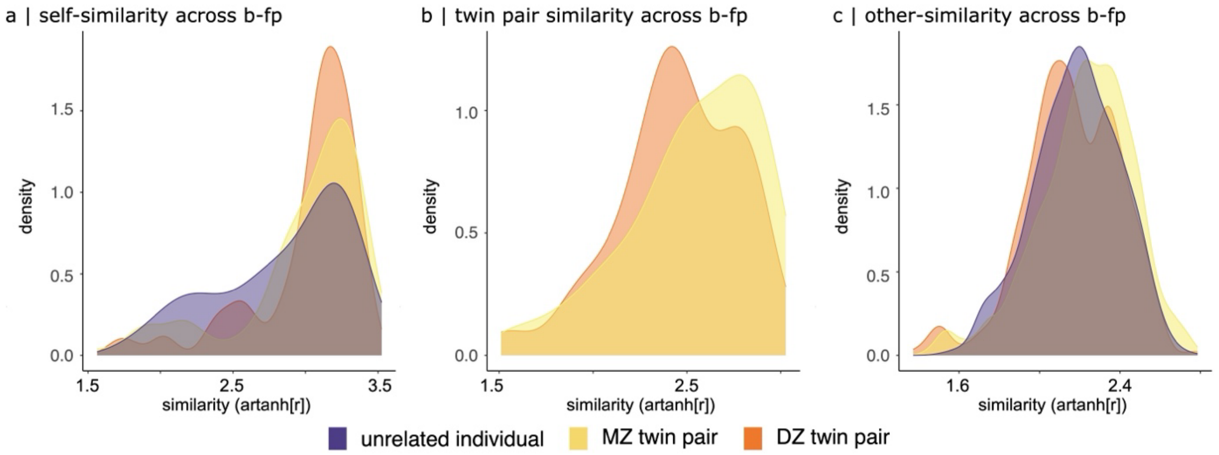

Figure S3: Self-, twin-pair, and other-similarity between neurophysiological profiles.

a) Self-similarity did not significantly differ between monozygotic (MZ) and dizygotic (DZ) twin pairs. Unrelated individuals exhibited a similar mean self-similarity but greater inter-individual variability (higher standard deviation).

b) Neurophysiological profiles (1–150 Hz) within MZ twin pairs were more similar than those of DZ twin pairs, and twin-pair similarity was greater than other-similarity.

c) Similarity between neurophysiological profiles of unrelated individuals (other-similarity) did not significantly differ across the three groups (MZ & DZ twins, and non-twin individuals).

Legend: b-fp, brain-fingerprint.

The Matching Between the Neurophysiological Profiles of Monozygotic Twins Is Not Driven by Anatomy

We tested whether the matching between the neurophysiological profiles of MZ twins may have been driven by similarities in their brain anatomy. The anatomical features extracted included: the Number of vertices, surface area, gray matter volume, mean cortical thickness, s.d. of cortical thickness, mean curvature, Gaussian curvature, folding index, and the curvature index (*91*). The brain structural features of MZ siblings extracted from *Freesurfer* (*92*) were indeed more similar than those of DZ twins or unrelated participants (see Methods): they showed a high correlation between MZ siblings (r= 0.99), and lower for DZ twin pairs (r= 0.93). The most heritable brain anatomical features the most heritable were related to the curvature (h= 1.75) and thickness (h= 1.75) of the cortex.

To assess the extent to which these brain structural features contributed to the heritability of neurophysiological profiles, we estimated the linear correlation between the matching of neurophysiological profiles of twin siblings and the similarity of their respective brain anatomical features (see Methods). We matched twin pairs using all 9 features for every parcel of the Desikan-Killiany atlas. These relationships were not statistically significant between MZ siblings (r= 0.32, p= 0.06) nor between DZ siblings (r= -0.22, p= 0.32; see Table S2). The outcome was similar when we replicated this analysis for neurophysiological profiles derived from alpha-band (MZ: r= 0.31, p= 0.08; DZ: r= -0.17, p= 0.45) and beta-band (MZ: r= 0.32, p= 0.07; DZ: r= -0.15, p= 0.51) brain activity. Bayes factor analyses corroborated that there was little evidence for a relationship between similarities of structural and neurophysiological traits (Supplemental Table S2). To conclude, while brain curvature and cortical thickness are heritable brain phenotypes, they did not contribute significantly to individual differentiation based on their neurophysiological profiles.

	Pearson's r		BF_{10}	
	MZ	DZ	MZ	DZ
Anatomy vs. broadband neurophysiology	0.32	-0.22	1.65	0.68
Anatomy vs. alpha-band neurophysiology	0.31	-0.17	1.46	0.57
Anatomy vs. Beta-band neurophysiology	0.31	-0.15	1.60	0.54

Table S2: Pearson's Correlations Between the Similarity of Brain Anatomy and Neurophysiological Profiles.

We fit linear models relating the similarity of brain anatomical similarity and neurophysiological profiles derived from broadband (1-150 Hz), alpha band, and beta band activity between MZ and DZ twin siblings. There was little Bayes factor evidence (BF_{10}) of such a relationship.

Salient Features for Participant Differentiation are Heritable

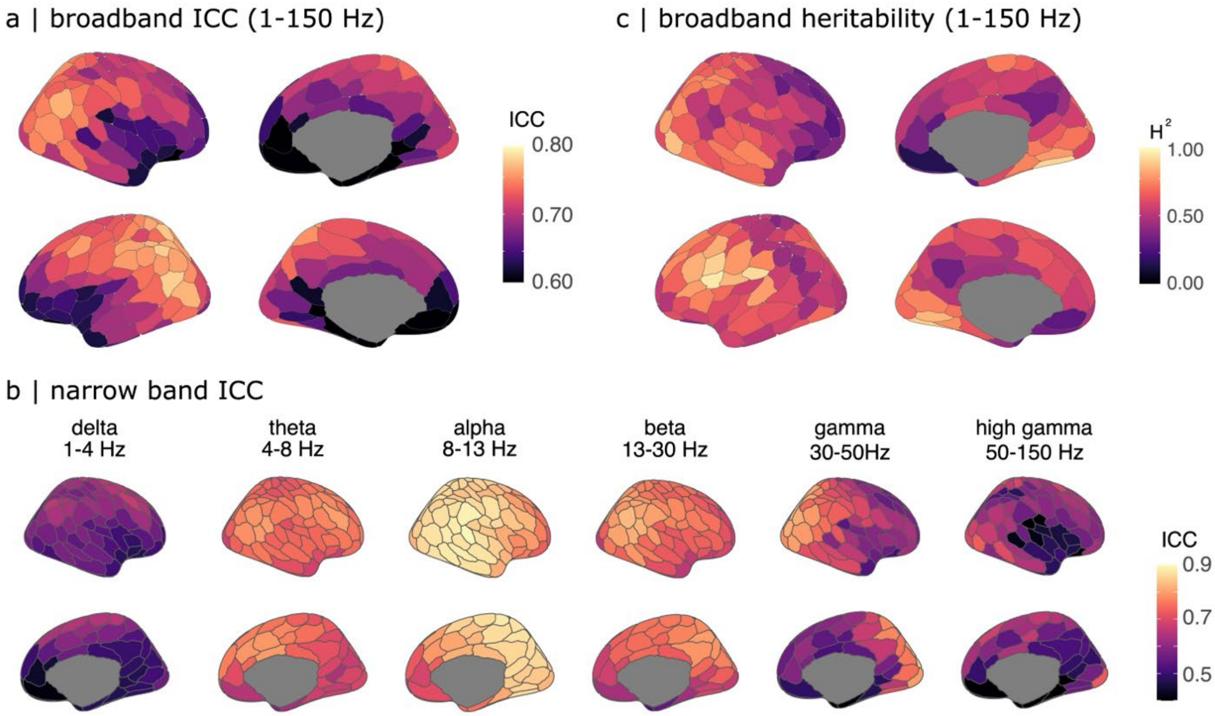

Figure S4. Salient Regions of Neurophysiological Profiles and Their Heritable Brain Phenotypes
(a & b) Topographic maps highlight the cortical regions with the most salient neurophysiological activity across all frequency bands (panel a) and per frequency band (panel b), as measured using intra-class correlation (ICC) statistics (see Main Text and Methods). These regions contribute most strongly to participant differentiation.

Note that the broadband (1-150 Hz) ICC topographic maps in panel (a) were obtained by averaging the ICC values across the six narrow frequency bands (delta, theta, alpha, beta, gamma, high gamma), ensuring equal weighting regardless of bandwidth differences.

(c) Topographic maps depicting the heritability of broadband neurophysiological activity (1-150 Hz). For frequency-specific maps of heritable neurophysiological traits, refer to Figure 1C in the main text.

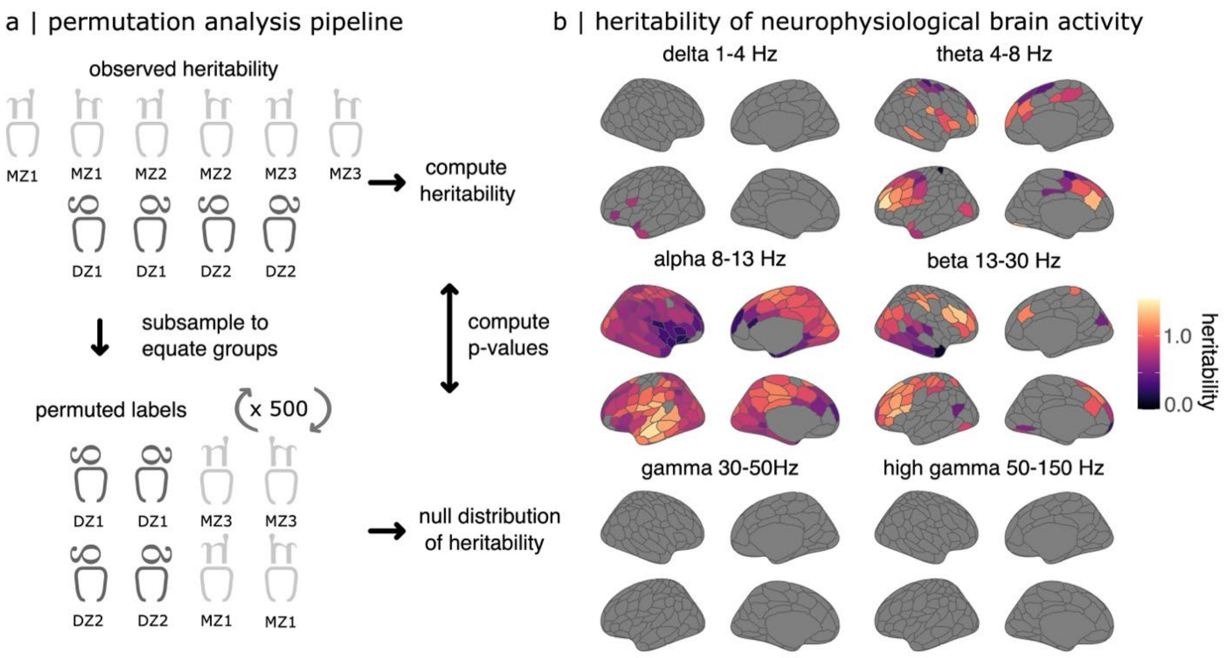

Figure S5. Significantly Heritable Neurophysiological Traits.

a) Analysis pipeline for assessing the statistical significance of heritability estimates. Heritability (H^2) was computed as the difference in neurophysiological profile concordance between monozygotic (MZ) and dizygotic (DZ) twin pairs. To generate a spatially realistic null distribution, MZ and DZ twin labels were randomly reassigned across 1000 iterations, after first subsampling MZ pairs to match the number of DZ pairs in each permutation. Spatial autocorrelation of the data was preserved throughout.

b) Results of the permutation analysis across frequency bands and cortical regions. Cortical maps show brain regions where heritability estimates significantly exceeded chance levels ($p_{FDR} < 0.05$). Significant heritability was observed in frontal regions for theta-band activity, across widespread cortical areas for alpha-band activity, and in frontal and parietal regions for beta-band activity.

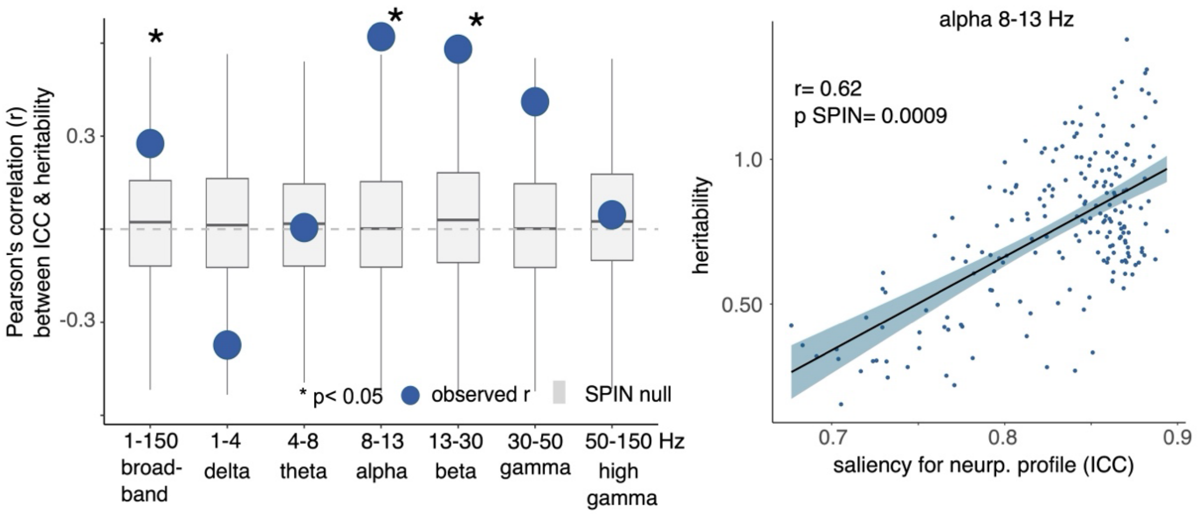

Figure S6: Differentiable Neurophysiological Traits are Heritable.
Left panel: Pearson's correlation between the neurophysiological traits that are the most salient for individual differentiation and their heritability, for each tested frequency band of electrophysiology. The blue dots indicate the correlation statistics and the boxplots depict the null distributions obtained by spin-test permutations.
Right panel: Scatter plot of best linear model relating the heritability and saliency of alpha-band neurophysiological traits. Each dot represents a region of the Schaefer-200 atlas. The saliency of the alpha-band neurophysiological traits for individual differentiation is linearly related to their heritability. This further demonstrates the genetic influence on neurophysiological traits.

Psychological Processes and Differentiation

We assessed the similarity in gene-expression brain score between the outcomes of the gene-differentiation and gene-psychological processes PLS analyses. We found strong linear relationships between the identified gene scores and the PLS loadings (The Gene-Differentiation Gradient Correlates with Psychological Processes).

We further tested whether the outcome of the PLS analysis for psychological processes (see Main Text) covaried with individual differentiation. We anticipated such a relationship as both psychological processes and individual differentiation covary with similar gene expression signatures. The PLS of psychological-process and individual differentiation featured a single significant latent variable (p= 0.002) that explained 87.0% of the covariance between these variables (85.2% covariance explained, p_{SPIN}= 0.002, 95% CI = [54.24., 87.37]). The ICC loadings and psychological process term loadings were linearly related to the loadings obtained from the previously reported PLS analysis (ICC loadings similarity, r=0.78; term loading similarity, r= 0.92).

Supplementary Data:

Supplemental Data 1: Negative gene set used for the gene ontology analysis. Table of the gene name, EntrezID, and PLS loading of the negative set of genes used in the GO analysis.

Supplemental Data 2: Positive gene set used for the gene ontology analysis. Table of the gene name, EntrezID, and PLS loading of the positive set of genes used in the GO analysis.

Supplemental Data 3: Results of the gene ontology analysis for the negative gene set. Table of the GO results for the negative gene set. Rows correspond to biological processes from the GO analysis, with their corresponding fold enrichment, p-value, and the corresponding genes that make up that GO category.

Supplemental Data 4: Results of the gene ontology analysis for the positive gene set. Table of the GO results for the positive gene set. Rows correspond to biological processes from the GO analysis, with their corresponding fold enrichment, p-value, and the corresponding genes that make up that GO category.

REFERENCES AND NOTES

1. E. S. Finn, X. Shen, D. Scheinost, M. D. Rosenberg, J. Huang, M. M. Chun, X. Papademetris, R. T. Constable, Functional connectome fingerprinting: identifying individuals using patterns of brain connectivity. *Nat. Neurosci.* **18**, 1664–1671 (2015).

2. J. da Silva Castanheira, H. D. Orozco Perez, B. Misic, S. Baillet, Brief segments of neurophysiological activity enable individual differentiation. *Nat. Commun.* **12**, 5713 (2021).

3. E. Amico, J. Goñi, The quest for identifiability in human functional connectomes. *Sci. Rep.* **8**, 8254 (2018).

4. T. Kaufmann, D. Alnæs, C. L. Brandt, F. Bettella, S. Djurovic, O. A. Andreassen, L. T. Westlye, Stability of the Brain Functional Connectome Fingerprint in Individuals With Schizophrenia. *JAMA Psychiatry* **75**, 749–751 (2018).

5. T. Kaufmann, D. Alnæs, N. T. Doan, C. L. Brandt, O. A. Andreassen, L. T. Westlye, Delayed stabilization and individualization in connectome development are related to psychiatric disorders. *Nat. Neurosci.* **20**, 513–515 (2017).

6. P. Sorrentino, R. Rucco, A. Lardone, M. Liparoti, E. Troisi Lopez, C. Cavaliere, A. Soricelli, V. Jirsa, G. Sorrentino, E. Amico, Clinical connectome fingerprints of cognitive decline. *Neuroimage* **238**, 118253 (2021).

7. E. Troisi Lopez, R. Minino, M. Liparoti, A. Polverino, A. Romano, R. De Micco, F. Lucidi, A. Tessitore, E. Amico, G. Sorrentino, V. Jirsa, P. Sorrentino, Fading of brain network fingerprint in Parkinson's disease predicts motor clinical impairment. *Hum. Brain Mapp.* **44**, 1239–1250 (2023).

8. J. da Silva Castanheira, A. I. Wiesman, J. Y. Hansen, B. Misic, S. Baillet, J. Breitner, J. Poirier, P. Bellec, V. Bohbot, M. Chakravarty, The neurophysiological brain-fingerprint of Parkinson's disease. *EBioMedicine* **105**, 105201 (2024).

9. Z. Fu, J. Liu, M. S. Salman, J. Sui, V. D. Calhoun, Functional connectivity uniqueness and variability? Linkages with cognitive and psychiatric problems in children. *Nat. Ment. Health* **1**, 956–970 (2023).

10. S. Stampacchia, S. Asadi, S. Tomczyk, F. Ribaldi, M. Scheffler, K.-O. Lövblad, M. Pievani, A. B. Fall, M. G. Preti, P. G. Unschuld, D. Van De Ville, O. Blanke, G. B. Frisoni, V. Garibotto, E. Amico, Fingerprints of brain disease: connectome identifiability in Alzheimer's disease. *Commun. Biol.* **7**, 1–16 (2024).

11. D. S. Falconer, The inheritance of liability to certain diseases, estimated from the incidence among relatives. *Ann. Hum. Genet.* **29**, 51–76 (1965).

12. F. Pizzagalli, G. Auzias, Q. Yang, S. R. Mathias, J. Faskowitz, J. D. Boyd, A. Amini, D. Rivière, K. L. McMahon, G. I. de Zubicaray, N. G. Martin, J.-F. Mangin, D. C. Glahn, J. Blangero, M. J. Wright, P. M. Thompson, P. Kochunov, N. Jahanshad, The reliability and heritability of cortical folds and their genetic correlations across hemispheres. *Commun. Biol.* **3**, 1–12 (2020).

13. D. Posthuma, E. J. C. de Geus, E. J. C. M. Mulder, D. J. A. Smit, D. I. Boomsma, C. J. Stam, Genetic components of functional connectivity in the brain: The heritability of synchronization likelihood. *Hum. Brain Mapp.* **26**, 191–198 (2005).

14. J. E. Schmitt, A. Raznahan, S. Liu, M. C. Neale, The heritability of cortical folding: Evidence from the Human Connectome Project. *Cereb. Cortex* **31**, 702–715 (2020).

15. P. H. Lee, J. T. Baker, A. J. Holmes, N. Jahanshad, T. Ge, J.-Y. Jung, Y. Cruz, D. S. Manoach, D. P. Hibar, J. Faskowitz, K. L. McMahon, G. I. de Zubicaray, N. H. Martin, M. J. Wright, D. Öngür, R. Buckner, J. Roffman, P. M. Thompson, J. W. Smoller, Partitioning heritability analysis reveals a shared genetic basis of brain anatomy and schizophrenia. *Mol. Psychiatry* **21**, 1680–1689 (2016).

16. C. M. Smit, M. J. Wright, N. K. Hansell, G. M. Geffen, N. G. Martin, Genetic variation of individual alpha frequency (IAF) and alpha power in a large adolescent twin sample. *Int. Organ. Psychophysiol.* **61**, 235–243 (2006).

17. E. Salmela, H. Renvall, J. Kujala, O. Hakosalo, M. Illman, M. Vihla, E. Leinonen, R. Salmelin, J. Kere, Evidence for genetic regulation of the human parieto-occipital 10-Hz rhythmic activity. *Eur. J. Neurosci.* **44**, 1963–1971 (2016).

18. B. P. Zietsch, J. L. Hansen, N. K. Hansell, G. M. Geffen, N. G. Martin, M. J. Wright, Common and specific genetic influences on EEG power bands delta, theta, alpha, and beta. *Biol. Psychol.* **75**, 154–164 (2007).

19. R. F. Betzel, J. D. Medaglia, A. E. Kahn, J. Soffer, D. R. Schonhaut, D. S. Bassett, Structural, geometric and genetic factors predict interregional brain connectivity patterns probed by electrocorticography. *Nat. Biomed. Eng.* **3**, 902–916 (2019).

20. J. Richiardi, A. Altmann, A.-C. Milazzo, C. Chang, M. M. Chakravarty, T. Banaschewski, G. J. Barker, A. L. W. Bokde, U. Bromberg, C. Büchel, P. Conrod, M. Fauth-Bühler, H. Flor, V. Frouin, J. Gallinat, H. Garavan, P. Gowland, A. Heinz, H. Lemaître, K. F. Mann, J.-L. Martinot, F. Nees, T. Paus, Z. Pausova, M. Rietschel, T. W. Robbins, M. N. Smolka, R. Spanagel, A. Ströhle, G. Schumann, M. Hawrylycz, J.-B. Poline, M. D. Greicius, IMAGEN CONSORTIUM, Correlated gene expression supports synchronous activity in brain networks. *Science* **348**, 1241–1244 (2015).

21. B. D. Fulcher, J. D. Murray, V. Zerbi, X.-J. Wang, Multimodal gradients across mouse cortex. *Proc. Natl. Acad. Sci. U.S.A.* **116**, 4689–4695 (2019).

22. I. Zwir, J. Arnedo, C. Del-Val, L. Pulkki-Råback, B. Konte, S. S. Yang, R. Romero-Zaliz, M. Hintsanen, K. M. Cloninger, D. Garcia, D. M. Svrakic, S. Rozsa, M. Martinez, L.-P. Lyytikäinen, I. Giegling, M. Kähönen, H. Hernandez-Cuervo, I. Seppälä, E. Raitoharju, G. A. de Erausquin, O. Raitakari, D. Rujescu, T. T. Postolache, J. Sung, L. Keltikangas-Järvinen, T. Lehtimäki, C. R. Cloninger, Uncovering the complex genetics of human character. *Mol. Psychiatry* **25**, 2295–2312 (2020).

23. R. A. Power, M. Pluess, Heritability estimates of the Big Five personality traits based on common genetic variants. *Transl. Psychiatry* **5**, e604 (2015).

24. J. Mollon, E. E. M. Knowles, S. R. Mathias, R. Gur, J. M. Peralta, D. J. Weiner, E. B. Robinson, R. E. Gur, J. Blangero, L. Almasy, D. C. Glahn, Genetic influence on cognitive development between childhood and adulthood. *Mol. Psychiatry* **26**, 656–665 (2021).

25. T. J. Bouchard, M. McGue, Genetic and environmental influences on human psychological differences. *J. Neurobiol.* **54**, 4–45 (2003).

26. C. Haworth, M. Wright, M. Luciano, N. Martin, E. de Geus, C. van Beijsterveldt, M. Bartels, D. Posthuma, D. Boomsma, O. Davis, Y. Kovas, R. Corley, J. DeFries, J. Hewitt, R. Olson, S.-A. Rhea, S. Wadsworth, W. Iacono, M. McGue, L. Thompson, S. Hart, S. Petrill, D. Lubinski, R. Plomin, The heritability of general cognitive ability increases linearly from childhood to young adulthood. *Mol. Psychiatry* **15**, 1112–1120 (2010).

27. S. Baillet, Magnetoencephalography for brain electrophysiology and imaging. *Nat. Neurosci.* **20**, 327–339 (2017).

28. A. J. Mayhew, D. Meyre, Assessing the Heritability of Complex Traits in Humans: Methodological Challenges and Opportunities. *Curr. Genomics* **18**, 332–340 (2017).

29. D. C. Van Essen, K. Ugurbil, E. Auerbach, D. Barch, T. E. J. Behrens, R. Bucholz, A. Chang, L. Chen, M. Corbetta, S. W. Curtiss, S. Della Penna, D. Feinberg, M. F. Glasser, N. Harel, A. C. Heath, L. Larson-Prior, D. Marcus, G. Michalareas, S. Moeller, R. Oostenveld, S. E. Petersen, F. Prior, B. L. Schlaggar, S. M. Smith, A. Z. Snyder, J. Xu, E. Yacoub, The Human Connectome Project: A data acquisition perspective. *Neuroimage* **62**, 2222–2231 (2012).

30. J. da Silva Castanheira, A. I. Wiesman, J. Y. Hansen, B. Misic, S. Baillet, PREVENT-AD Research Group, Quebec Parkinson Network, Neurophysiological brain-fingerprints of motor and cognitive decline in Parkinson’s disease. medRxiv [Preprint] (2023); https://doi.org/10.1101/2023.02.03.23285441.

31. J. B. Burt, M. Demirtaş, W. J. Eckner, N. M. Navejar, J. L. Ji, W. J. Martin, A. Bernacchia, A. Anticevic, J. D. Murray, Hierarchy of transcriptomic specialization across human cortex captured by structural neuroimaging topography. *Nat. Neurosci.* **21**, 1251–1259 (2018).

32. J. Y. Hansen, R. D. Markello, J. W. Vogel, J. Seidlitz, D. Bzdok, B. Misic, Mapping gene transcription and neurocognition across human neocortex. *Nat. Hum. Behav.* **5**, 1240–1250 (2021).

33. M. J. Hawrylycz, E. S. Lein, A. L. Guillozet-Bongaarts, E. H. Shen, L. Ng, J. A. Miller, L. N. van de Lagemaat, K. A. Smith, A. Ebbert, Z. L. Riley, C. Abajian, C. F. Beckmann, A. Bernard, D. Bertagnolli, A. F. Boe, P. M. Cartagena, M. M. Chakravarty, M. Chapin, J. Chong, R. A. Dalley, B. D. Daly, C. Dang, S. Datta, N. Dee, T. A. Dolbeare, V. Faber, D. Feng, D. R. Fowler, J. Goldy, B. W. Gregor, Z. Haradon, D. R. Haynor, J. G. Hohmann, S. Horvath, R. E. Howard, A. Jeromin, J. M. Jochim, M. Kinnunen, C. Lau, E. T. Lazarz, C. Lee, T. A. Lemon, L. Li, Y. Li, J. A. Morris, C. C. Overly, P. D. Parker, S. E. Parry, M. Reding, J. J. Royall, J. Schulkin, P. A. Sequeira, C. R. Slaughterbeck, S. C. Smith, A. J. Sodt, S. M. Sunkin, B. E. Swanson, M. P. Vawter, D. Williams, P. Wohnoutka, H. R. Zielke, D. H. Geschwind, P. R. Hof, S. M. Smith, C. Koch, S. G. N. Grant, A. R. Jones, An anatomically comprehensive atlas of the adult human brain transcriptome. *Nature* **489**, 391–399 (2012).

34. M. Hawrylycz, J. A. Miller, V. Menon, D. Feng, T. Dolbeare, A. L. Guillozet-Bongaarts, A. G. Jegga, B. J. Aronow, C.-K. Lee, A. Bernard, M. F. Glasser, D. L. Dierker, J. Menche, A. Szafer, F. Collman, P. Grange, K. A. Berman, S. Mihalas, Z. Yao, L. Stewart, A.-L. Barabási, J. Schulkin, J. Phillips, L. Ng, C. Dang, D. R. Haynor, A. Jones, D. C. Van Essen, C. Koch, E. Lein, Canonical genetic signatures of the adult human brain. *Nat. Neurosci.* **18**, 1832–1844 (2015).

35. R. D. Markello, A. Arnatkeviciute, J.-B. Poline, B. D. Fulcher, A. Fornito, B. Misic, Standardizing workflows in imaging transcriptomics with the abagen toolbox. *eLife* **10**, e72129 (2021).

36. S. X. Ge, D. Jung, R. Yao, ShinyGO: a graphical gene-set enrichment tool for animals and plants. *Bioinformatics* **36**, 2628–2629 (2020).

37. Y. Zhang, S. A. Sloan, L. E. Clarke, C. Caneda, C. A. Plaza, P. D. Blumenthal, H. Vogel, G. K. Steinberg, M. S. B. Edwards, G. Li, J. A. Duncan, S. H. Cheshier, L. M. Shuer, E. F. Chang, G. A. Grant, M. G. H. Gephart, B. A. Barres, Purification and Characterization of

Progenitor and Mature Human Astrocytes Reveals Transcriptional and Functional Differences with Mouse. *Neuron* **89**, 37–53 (2016).

38. B. B. Lake, S. Chen, B. C. Sos, J. Fan, G. E. Kaeser, Y. C. Yung, T. E. Duong, D. Gao, J. Chun, P. V. Kharchenko, K. Zhang, Integrative single-cell analysis of transcriptional and epigenetic states in the human adult brain. *Nat. Biotechnol.* **36**, 70–80 (2018).

39. N. Habib, I. Avraham-Davidi, A. Basu, T. Burks, K. Shekhar, M. Hofree, S. R. Choudhury, F. Aguet, E. Gelfand, K. Ardlie, D. A. Weitz, O. Rozenblatt-Rosen, F. Zhang, A. Regev, Massively parallel single-nucleus RNA-seq with DroNc-seq. *Nat. Methods* **14**, 955–958 (2017).

40. S. Darmanis, S. A. Sloan, Y. Zhang, M. Enge, C. Caneda, L. M. Shuer, M. G. Hayden Gephart, B. A. Barres, S. R. Quake, A survey of human brain transcriptome diversity at the single cell level. *Proc. Natl. Acad. Sci. U.S.A* **112**, 7285–7290 (2015).

41. M. Li, G. Santpere, Y. Imamura Kawasawa, O. V. Evgrafov, F. O. Gulden, S. Pochareddy, S. M. Sunkin, Z. Li, Y. Shin, Y. Zhu, A. M. M. Sousa, D. M. Werling, R. R. Kitchen, H. J. Kang, M. Pletikos, J. Choi, S. Muchnik, X. Xu, D. Wang, B. Lorente-Galdos, S. Liu, P. Giusti-Rodríguez, H. Won, C. A. de Leeuw, A. F. Pardiñas, BrainSpan Consortium, PsychENCODE Consortium, PsychENCODE Developmental Subgroup, M. Hu, F. Jin, Y. Li, M. J. Owen, M. C. O'Donovan, J. T. R. Walters, D. Posthuma, M. A. Reimers, P. Levitt, D. R. Weinberger, T. M. Hyde, J. E. Kleinman, D. H. Geschwind, M. J. Hawrylycz, M. W. State, S. J. Sanders, P. F. Sullivan, M. B. Gerstein, E. S. Lein, J. A. Knowles, N. Sestan, Integrative functional genomic analysis of human brain development and neuropsychiatric risks. *Science* **362**, eaat7615 (2018).

42. A. T. McKenzie, M. Wang, M. E. Hauberg, J. F. Fullard, A. Kozlenkov, A. Keenan, Y. L. Hurd, S. Dracheva, P. Casaccia, P. Roussos, B. Zhang, Brain Cell Type Specific Gene Expression and Co-expression Network Architectures. *Sci. Rep.* **8**, 8868 (2018).

43. S. Baillet, J. C. Mosher, R. M. Leahy, Electromagnetic brain mapping. *IEEE Signal Process. Mag.* **18**, 14–30 (2001).

44. M. Hämäläinen, R. Hari, R. J. Ilmoniemi, J. Knuutila, O. V. Lounasmaa, Magnetoencephalography—theory, instrumentation, and applications to noninvasive studies of the working human brain. *Rev. Mod. Phys.* **65**, 413–497 (1993).

45. M. D. Rosenberg, E. S. Finn, D. Scheinost, R. T. Constable, M. M. Chun, Characterizing Attention with Predictive Network Models. *Trends Cogn. Sci.* **21**, 290–302 (2017).

46. M. D. Rosenberg, D. Scheinost, A. S. Greene, E. W. Avery, Y. H. Kwon, E. S. Finn, R. Ramani, M. Qiu, R. T. Constable, M. M. Chun, Functional connectivity predicts changes in attention observed across minutes, days, and months. *Proc. Natl. Acad. Sci. U.S.A.* **117**, 3797–3807 (2020).

47. T. Yarkoni, R. A. Poldrack, T. E. Nichols, D. C. Van Essen, T. D. Wager, Large-scale automated synthesis of human functional neuroimaging data. *Nat. Methods* **8**, 665–670 (2011).

48. D. A. Briley, E. M. Tucker-Drob, Comparing the Developmental Genetics of Cognition and Personality over the Lifespan. *J. Pers.* **85**, 51–64 (2017).

49. J. A. Miller, S.-L. Ding, S. M. Sunkin, K. A. Smith, L. Ng, A. Szafer, A. Ebbert, Z. L. Riley, J. J. Royall, K. Aiona, J. M. Arnold, C. Bennet, D. Bertagnolli, K. Brouner, S. Butler, S. Caldejon, A. Carey, C. Cuhaciyan, R. A. Dalley, N. Dee, T. A. Dolbeare, B. A. C. Facer, D. Feng, T. P. Fliss, G. Gee, J. Goldy, L. Gourley, B. W. Gregor, G. Gu, R. E. Howard, J. M. Jochim, C. L. Kuan, C. Lau, C.-K. Lee, F. Lee, T. A. Lemon, P. Lesnar, B. McMurray, N. Mastan, N. Mosqueda, T. Naluai-Cecchini, N.-K. Ngo, J. Nyhus, A. Oldre, E. Olson, J. Parente, P. D. Parker, S. E. Parry, A. Stevens, M. Pletikos, M. Reding, K. Roll, D. Sandman, M. Sarreal, S. Shapouri, N. V. Shapovalova, E. H. Shen, N. Sjoquist, C. R. Slaughterbeck, M. Smith, A. J. Sodt, D. Williams, L. Zöllei, B. Fischl, M. B. Gerstein, D. H. Geschwind, I. A. Glass, M. J. Hawrylycz, R. F. Hevner, H. Huang, A. R. Jones, J. A. Knowles, P. Levitt, J. W. Phillips, N. Šestan, P. Wohnoutka, C. Dang, A. Bernard, J. G. Hohmann, E. S. Lein, Transcriptional landscape of the prenatal human brain. *Nature* **508**, 199–206 (2014).

50. E. Sareen, S. Zahar, D. V. D. Ville, A. Gupta, A. Griffa, E. Amico, Exploring MEG brain fingerprints: Evaluation, pitfalls, and interpretations. *Neuroimage* **240**, 118331 (2021).

51. J. da S. Castanheira, A. I. Wiesman, M. J. Taylor, S. Baillet, The Lifespan Evolution of Individualized Neurophysiological Traits. *bioRxiv* [Preprint] (2024). https://doi.org/10.1101/2024.11.27.624077.

52. J. Seidlitz, A. Nadig, S. Liu, R. A. I. Bethlehem, P. E. Vértes, S. E. Morgan, F. Váša, R. Romero-Garcia, F. M. Lalonde, L. S. Clasen, J. D. Blumenthal, C. Paquola, B. Bernhardt, K. Wagstyl, D. Polioudakis, L. de la Torre-Ubieta, D. H. Geschwind, J. C. Han, N. R. Lee, D. G. Murphy, E. T. Bullmore, A. Raznahan, Transcriptomic and cellular decoding of regional brain vulnerability to neurogenetic disorders. *Nat. Commun.* **11**, 3358 (2020).

53. F. M. Krienen, B. T. T. Yeo, T. Ge, R. L. Buckner, C. C. Sherwood, Transcriptional profiles of supragranular-enriched genes associate with corticocortical network architecture in the human brain. *Proc. Natl. Acad. Sci. U.S.A.* **113**, E469–E478 (2016).

54. B. D. Fulcher, A. Fornito, A transcriptional signature of hub connectivity in the mouse connectome. *Proc. Natl. Acad. Sci. U.S.A.* **113**, 1435–1440 (2016).

55. K. J. Whitaker, P. E. Vértes, R. Romero-Garcia, F. Váša, M. Moutoussis, G. Prabhu, N. Weiskopf, M. F. Callaghan, K. Wagstyl, T. Rittman, R. Tait, C. Ooi, J. Suckling, B. Inkster, P. Fonagy, R. J. Dolan, P. B. Jones, I. M. Goodyer, the NSPN Consortium, E. T. Bullmore, Adolescence is associated with genomically patterned consolidation of the hubs of the human brain connectome. *Proc. Natl. Acad. Sci. U.S.A.* **113**, 9105–9110 (2016).

56. A. F. Alexander-Bloch, A. Raznahan, S. N. Vandekar, J. Seidlitz, Z. Lu, S. R. Mathias, E. Knowles, J. Mollon, A. Rodrigue, J. E. Curran, H. H. H. Görring, T. D. Satterthwaite, R. E. Gur, D. S. Bassett, G. D. Hoftman, G. Pearlson, R. T. Shinohara, S. Liu, P. T. Fox, L. Almasy, J. Blangero, D. C. Glahn, Imaging local genetic influences on cortical folding. *Proc. Natl. Acad. Sci. U.S.A.* **117**, 7430–7436 (2020).

57. R. D. Markello, J. Y. Hansen, Z.-Q. Liu, V. Bazinet, G. Shafiei, L. E. Suárez, N. Blostein, J. Seidlitz, S. Baillet, T. D. Satterthwaite, M. M. Chakravarty, A. Raznahan, B. Misic, neuromaps: structural and functional interpretation of brain maps. *Nat. Methods* **19**, 1472–1479 (2022).

58. Y. Fu, Z. Ma, C. Hamilton, Z. Liang, X. Hou, X. Ma, X. Hu, Q. He, W. Deng, Y. Wang, L. Zhao, H. Meng, T. Li, N. Zhang, Genetic influences on resting-state functional networks: A twin study: Genetic Influences on Resting-State Functional Networks. *Hum. Brain Mapp.* **36**, 3959–3972 (2015).

59. A. Fornito, A. Zalesky, D. S. Bassett, D. Meunier, I. Ellison-Wright, M. Yucel, S. J. Wood, K. Shaw, J. O'Connor, D. Nertney, B. J. Mowry, C. Pantelis, E. T. Bullmore, Genetic Influences on Cost-Efficient Organization of Human Cortical Functional Networks. *J. Neurosci.* **31**, 3261–3270 (2011).

60. G. Winterer, R. Mahlberg, M. N. Smolka, J. Samochowiec, M. Ziller, H.-P. Rommelspacher, W. M. Herrmann, L. G. Schmidt, T. Sander, Association Analysis of Exonic Variants of the GABAB-Receptor Gene and Alpha Electroencephalogram Voltage in Normal Subjects and Alcohol-Dependent Patients. *Behav. Genet.* **33**, 7–15 (2003).

61. J. Y. Hansen, G. Shafiei, R. D. Markello, K. Smart, S. M. L. Cox, M. Nørgaard, V. Beliveau, Y. Wu, J.-D. Gallezot, É. Aumont, S. Servaes, S. G. Scala, J. M. DuBois, G. Wainstein, G. Bezgin, T. Funck, T. W. Schmitz, R. N. Spreng, M. Galovic, M. J. Koepp, J. S. Duncan, J. P. Coles, T. D. Fryer, F. I. Aigbirhio, C. J. McGinnity, A. Hammers, J.-P. Soucy, S. Baillet, S. Guimond, J. Hietala, M.-A. Bedard, M. Leyton, E. Kobayashi, P. Rosa-Neto, M. Ganz, G. M. Knudsen, N. Palomero-Gallagher, J. M. Shine, R. E. Carson, L. Tuominen, A. Dagher, B. Misic, Mapping neurotransmitter systems to the structural and functional organization of the human neocortex. *Nat. Neurosci.* **25**, 1569–1581 (2022).

62. D. S. Margulies, S. S. Ghosh, A. Goulas, M. Falkiewicz, J. M. Huntenburg, G. Langs, G. Bezgin, S. B. Eickhoff, F. X. Castellanos, M. Petrides, E. Jefferies, J. Smallwood, Situating the default-mode network along a principal gradient of macroscale cortical organization. *Proc. Natl. Acad. Sci. U.S.A.* **113**, 12574–12579 (2016).

63. K. Wagstyl, A. Raznahan, Converging cortical axes. *Nat. Neurosci.* **28**, 8–10 (2025).

64. V. J. Sydnor, B. Larsen, D. S. Bassett, A. Alexander-Bloch, D. A. Fair, C. Liston, A. P. Mackey, M. P. Milham, A. Pines, D. R. Roalf, J. Seidlitz, T. Xu, A. Raznahan, T. D.

Satterthwaite, Neurodevelopment of the association cortices: Patterns, mechanisms, and implications for psychopathology. *Neuron* **109**, 2820–2846 (2021).

65. T. Kaufmann, D. Alnæs, C. L. Brandt, F. Bettella, S. Djurovic, O. A. Andreassen, L. T. Westlye, Stability of the Brain Functional Connectome Fingerprint in Individuals With Schizophrenia. *JAMA Psychiatry* **75**, 749–751 (2018).

66. K. Thuwal, A. Banerjee, D. Roy, Aperiodic and Periodic Components of Ongoing Oscillatory Brain Dynamics Link Distinct Functional Aspects of Cognition across Adult Lifespan. *eNeuro* **8**, ENEURO.0224-21.2021 (2021).

67. B. Voytek, M. A. Kramer, J. Case, K. Q. Lepage, Z. R. Tempesta, R. T. Knight, A. Gazzaley, Age-Related Changes in 1/f Neural Electrophysiological Noise. *J. Neurosci.* **35**, 13257–13265 (2015).

68. B. Voytek, R. T. Knight, Dynamic Network Communication as a Unifying Neural Basis for Cognition, Development, Aging, and Disease. *Biol. Psychiatry* **77**, 1089–1097 (2015).

69. C.-H. Cheng, P.-Y. S. Chan, S. Baillet, Y.-Y. Lin, Age-Related Reduced Somatosensory Gating Is Associated with Altered Alpha Frequency Desynchronization. *Neural Plast.* **2015**, e302878 (2015).

70. T. Hinault, S. Baillet, S. M. Courtney, Age-related changes of deep-brain neurophysiological activity. *Cereb. Cortex* **33**, 3960–3968 (2023).

71. L. Li, Y. Wei, J. Zhang, J. Ma, Y. Yi, Y. Gu, L. M. W. Li, Y. Lin, Z. Dai, Gene expression associated with individual variability in intrinsic functional connectivity. *Neuroimage* **245**, 118743 (2021).

72. G. Shafiei, S. Baillet, B. Misic, Human electromagnetic and haemodynamic networks systematically converge in unimodal cortex and diverge in transmodal cortex. *PLOS Biol.* **20**, e3001735 (2022).

73. J. C. Pang, K. M. Aquino, M. Oldehinkel, P. A. Robinson, B. D. Fulcher, M. Breakspear, A. Fornito, Geometric constraints on human brain function. *Nature* **618**, 566–574 (2023).

74. T. Sarwar, Y. Tian, B. T. T. Yeo, K. Ramamohanarao, A. Zalesky, Structure-function coupling in the human connectome: A machine learning approach. *Neuroimage* **226**, 117609 (2021).

75. L. E. Liharska, Y. J. Park, K. Ziafat, L. Wilkins, H. Silk, L. M. Linares, R. C. Thompson, E. Vornholt, B. Sullivan, V. Cohen, P. Kota, C. Feng, E. Cheng, J. S. Johnson, M.-K. Rieder, J. Huang, J. Scarpa, J. Polanco, E. Moya, A. Hashemi, M. A. Levin, G. N. Nadkarni, R. Sebra, J. Crary, E. E. Schadt, N. D. Beckmann, B. H. Kopell, A. W. Charney, A study of gene expression in the living human brain. medRxiv [Preprint] (2023). https://doi.org/10.1101/2023.04.21.23288916.

76. J. S. Elam, M. F. Glasser, M. P. Harms, S. N. Sotiropoulos, J. L. R. Andersson, G. C. Burgess, S. W. Curtiss, R. Oostenveld, L. J. Larson-Prior, J.-M. Schoffelen, M. R. Hodge, E. A. Cler, D. M. Marcus, D. M. Barch, E. Yacoub, S. M. Smith, K. Ugurbil, D. C. Van Essen, The Human Connectome Project: A retrospective. *Neuroimage* **244**, 118543 (2021).

77. J. Gross, S. Baillet, G. R. Barnes, R. N. Henson, A. Hillebrand, O. Jensen, K. Jerbi, V. Litvak, B. Maess, R. Oostenveld, L. Parkkonen, J. R. Taylor, V. van Wassenhove, M. Wibral, J.-M. Schoffelen, Good practice for conducting and reporting MEG research. *Neuroimage* **65**, 349–363 (2013).

78. F. Tadel, S. Baillet, J. C. Mosher, D. Pantazis, R. M. Leahy, Brainstorm: A User-Friendly Application for MEG/EEG Analysis. *Comput. Intell. Neurosci.* **2011**, 1–13 (2011).

79. A. Schaefer, R. Kong, E. M. Gordon, T. O. Laumann, X.-N. Zuo, A. J. Holmes, S. B. Eickhoff, B. T. T. Yeo, Local-Global Parcellation of the Human Cerebral Cortex from Intrinsic Functional Connectivity MRI. *Cereb. Cortex* **28**, 3095–3114 (2018).

80. E. Amico, J. Goñi, Mapping hybrid functional-structural connectivity traits in the human connectome. *Netw. Neurosci.* **2**, 306–322 (2018).

81. A. Arnatkevičiūtė, B. D. Fulcher, A. Fornito, A practical guide to linking brain-wide gene expression and neuroimaging data. *Neuroimage* **189**, 353–367 (2019).

82. A. Krishnan, L. J. Williams, A. R. McIntosh, H. Abdi, Partial Least Squares (PLS) methods for neuroimaging: a tutorial and review. *Neuroimage* **56**, 455–475 (2011).

83. A. R. McIntosh, B. Mišić, Multivariate statistical analyses for neuroimaging data. *Annu. Rev. Psychol.* **64**, 499–525 (2013).

84. A. R. McIntosh, F. L. Bookstein, J. V. Haxby, C. L. Grady, Spatial pattern analysis of functional brain images using partial least squares. *Neuroimage* **3**, 143–157 (1996).

85. M. Ashburner, C. A. Ball, J. A. Blake, D. Botstein, H. Butler, J. M. Cherry, A. P. Davis, K. Dolinski, S. S. Dwight, J. T. Eppig, M. A. Harris, D. P. Hill, L. Issel-Tarver, A. Kasarskis, S. Lewis, J. C. Matese, J. E. Richardson, M. Ringwald, G. M. Rubin, G. Sherlock, Gene Ontology: tool for the unification of biology. *Nat. Genet.* **25**, 25–29 (2000).

86. P. D. Thomas, The Gene Ontology and the meaning of biological function. *Methods Mol. Biol. Clifton NJ* **1446**, 15 (2017).

87. B. D. Fulcher, A. Arnatkeviciute, A. Fornito, Overcoming false-positive gene-category enrichment in the analysis of spatially resolved transcriptomic brain atlas data. *Nat. Commun.* **12**, 2669 (2021).

88. R Core Team, R: A Langeuage and Environment for Statistical Computing (R Foundation for Statistical Computing, Vienna, Austria, 2022; https://www.R-project.org/).

89. R. D. Markello, B. Misic, Comparing spatial null models for brain maps. *Neuroimage* **236**, 118052 (2021).

90. F. Váša, B. Mišić, Null models in network neuroscience. *Nat. Rev. Neurosci.* **23**, 493–504 (2022).

91. B. Fischl, *FreeSurfer. NeuroImage* **62**, 774–781 (2012).

92. R. A. Poldrack, A. Kittur, D. Kalar, E. Miller, C. Seppa, Y. Gil, D. S. Parker, F. W. Sabb, R. M. Bilder, The Cognitive Atlas: Toward a Knowledge Foundation for Cognitive Neuroscience. *Front. Neuroinformatics* **5**, 17 (2011).

93. D. M. Werling, S. Pochareddy, J. Choi, J.-Y. An, B. Sheppard, M. Peng, Z. Li, C. Dastmalchi, G. Santpere, A. M. M. Sousa, A. T. N. Tebbenkamp, N. Kaur, F. O. Gulden, M. S. Breen, L. Liang, M. C. Gilson, X. Zhao, S. Dong, L. Klei, A. E. Cicek, J. D. Buxbaum, H. Adle-Biassette, J.-L. Thomas, K. A. Aldinger, D. R. O'Day, I. A. Glass, N. A. Zaitlen, M. E. Talkowski, K. Roeder, M. W. State, B. Devlin, S. J. Sanders, N. Sestan, Whole-Genome and RNA Sequencing Reveal Variation and Transcriptomic Coordination in the Developing Human Prefrontal Cortex. *Cell Rep.* **31**, 107489 (2020).
